# *Lactobacillus johnsonii* alleviates experimental colitis by restoring intestinal barrier function and reducing NET-mediated gut-liver inflammation

**DOI:** 10.1038/s42003-025-08679-4

**Published:** 2025-08-14

**Authors:** Hao-Yu Liu, Peng Yuan, Shicheng Li, Kennedy Jerry Ogamune, Xinyu Shi, Cuipeng Zhu, Wael Ennab, Ping Hu, Abdelkareem A. Ahmed, Yunzeng Zhang, In Ho Kim, Demin Cai

**Affiliations:** 1 College of Animal Science and Technology, Yangzhou University, Yangzhou, PR China; 2 Joint International Research Laboratory of Agricultural & Agri-Product Safety, The Ministry of Education of China, Yangzhou University, Yangzhou, PR China; 3 Jiangsu Key Laboratory of Zoonosis, Yangzhou University, Yangzhou, PR China; 4 Jiangsu Key Laboratory of Animal Genetic Breeding and Molecular Design, Yangzhou University, Yangzhou, PR China; 5 Department of Veterinary Biomedical Sciences, Botswana University of Agriculture and Natural Resources, Gaborone, Botswana; 6 Jiangsu Co-innovation Center for Prevention and Control of Important Animal Infectious Diseases and Zoonoses, Yangzhou University, Yangzhou, PR China; 7 Department of Animal Resource and Science, Dankook University, Cheonan, Republic of Korea

**Keywords:** Mucosal immunology, Microbiome, Chronic inflammation

## Abstract

Inflammatory bowel disease (IBD) remains a global health challenge linked to intestinal barrier disruption, microbiota dysbiosis, and immune dysregulation, though the interplay of these mechanisms remains poorly defined. Here, we investigated the therapeutic potential of *Lactobacillus johnsonii* N5 in a murine dextran sulfate sodium (DSS)-induced colitis model. Prophylactic N5 administration alleviated colitis symptoms (weight loss, colon shortening), reduced fecal and serum lipocalin-2 levels, and suppressed colonic pro-inflammatory cytokines (IL-1β, IL-6). N5 preserved microbial diversity, enhanced mucus secretion, and reinforced mucosal barrier integrity, preventing colitis onset. Therapeutically, N5 attenuated disease progression by downregulating IL-1β, IL-6, IL-8 expression, restoring *Lactobacillus* populations, and suppressing *Escherichia-Shigella* expansion, thereby reducing bacterial translocation and systemic inflammation. N5 promoted Ki67^+^ epithelial proliferation, accelerating mucosal repair. Mechanistically, N5 targeted neutrophil-mediated gut-liver injury, suppressing coagulation pathways in colon-liver transcriptomes, reducing hepatic lesions, platelet aggregation, CD162^+^ neutrophil recruitment, and H3cit ^+^ neutrophil extracellular trap (NET) formation. N5’s effects were partially recapitulated by DNase I in vivo and/or by its metabolites in vitro, suggesting its action involves metabolite-driven NET inhibition alongside DNase-like NET clearance. These findings illuminate N5’s dual role in IBD-prophylactic barrier fortification and therapeutic resolution of neutrophilic inflammation, and highlight its potential as a multifaceted probiotic therapy.

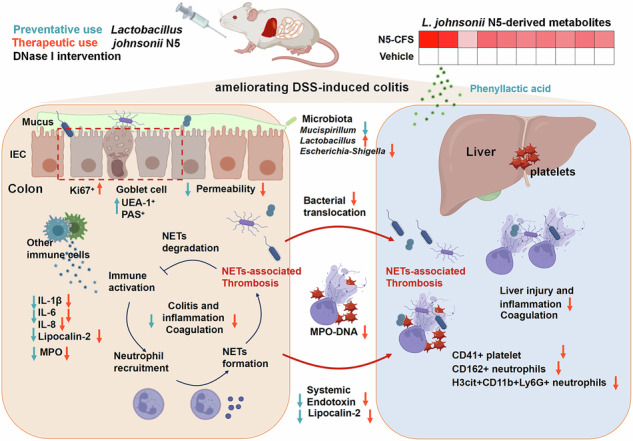

## Abstract (web summary)

*Lactobacillus johnsonii* N5 prevents colitis by fortifying the mucosal barrier and alleviates disease by inhibiting NETosis and gut-liver inflammation, promoting mucosal repair.

## Introduction

Inflammatory bowel disease (IBD), comprising Crohn’s disease (CD) and ulcerative colitis (UC), is a chronic gastrointestinal disorder with a rapidly rising incidence^1^. Despite significant global morbidity, therapeutic options for IBD remain limited. Current treatments—including anti-inflammatory corticosteroids, immunosuppressants, and biologics including anti-tumor necrosis factor-α (TNF-α) antibodies, are ineffective in a large proportion of patients^2^. A study of 376 IBD patients revealed that individuals with intestinal ulcerations exhibiting heightened neutrophil-chemoattractant activity linked to IL-1 signaling, rendering them non-responders to anti-TNF therapies^3^. The etiology of IBD is complex, involving diverse interplays between host physiology and environmental factors such as diet, genetic susceptibility, and microbial exposure, collectively driving a spectrum of heterogeneous inflammatory disorders^1,4^.

Gut homeostasis is maintained by a multi-layered mucosal barrier, including the epithelium with tight junctions and stratified mucus layers^5,6^. This barrier is intricately regulated through dynamic interactions between gut microbiota and host immune cells, which coordinate to suppress pathobiont overgrowth while tolerating commensal species^4,7^. Studies have identified significant compositional and metabolic changes in microbiota (dysbiosis) in IBD patients and murine colitis models, marked by reduced microbial diversity, altered short-chain fatty acid (SCFA) production and other metabolic shifts^6–8^. Notably, intestinal inflammation is not induced under germ-free conditions in these models, underscoring the microbiota’s indispensable role in IBD pathogenesis^9^. Conversely, IBD is characterized by depletion of commensal microbes, primarily Firmicutes, a predominant phylum critical for microbial metabolism, and overgrowth of pathobionts such as *Escherichia coli* and *Enterobacteriaceae* within the phylum Pseudomonadota (formerly known as Proteobacteria**)**^4,7,8^. This dysbiosis correlates with increased production of pro-inflammatory cytokines (e.g., IL-1β, TNF-α, and IL-6)^2,10^, leading to intestinal barrier disruption (“leaky gut”) and triggering aberrant immune responses that drive colitis^8,11^.

Neutrophils serve as the first line of immune defense against invading microbes and noxious agents, playing dual roles in the initiation, propagation, and resolution of gut inflammation^12–14^. The extent of neutrophil infiltration in inflamed colonic mucosa positively correlates with UC severity, suggesting their contribution to tissue damage^3,5^. Neutrophils employ antimicrobial mechanisms such as phagocytosis, degranulation, and reactive oxygen species (ROS) production to recruit immune cells and sequester pathogens^15–17^. They also form neutrophil extracellular traps (NETs) composed of DNA decorated with histones and proteases, through a process termed NETosis, to facilitate bacterial clearance^13,18,19^. However, excessive NET formation exacerbates inflammation, by directly injuring epithelial cells, promoting autoantigen exposure, and stimulating thrombosis^13,20,21^. Neutrophil from IBD patients exhibit heightened NETosis^22^, and elevated NETs levels are detected in blood^11,20,21^, biopsies^17,22,23^ and stool of patients with active IBD^13,20^, implicating dysregulated NETosis as a driver of chronic inflammation.

Neutrophil extracellular trap formation can be triggered by microorganisms (*E. coli*^24^, *Staphylococcus aureus*^18^, and *Citrobacter rotaries*^25^) and endogenous stimuli such as damage-associated molecular patterns (DAMPs) released during cell stress. Pathogen-activated receptor ligation drives NETosis, which some microbes exploit to reinforce biofilms, evade immune detection, or enhance systemic spread^25^. Conversely, probiotics like *Lactobacillus rhamnosus* GG (LGG) inhibit *S. aureus*-induced NET formation in murine bone marrow-derived neutrophils (BMDNs) and human myeloid cell cultures by modulating ROS and peptidylarginine deiminase 4 (PAD4) activity^26^, demonstrating that beneficial microbiota can actively suppress NETosis and its inflammatory consequences. Probiotics, defined as ‘Live microorganisms that, when administered in adequate amounts, confer a health benefit on the host'^27^, are emerging as UC therapies due to their ability to restore microbial balance and suppress hyperactive immunity^1^. Protective roles of *Lactobacillus* in colitis, including strengthening mucosal barriers via mucin upregulation, enhancing epithelial tight junctions, and reducing inflammation through IL-10 induction have been demonstrated by us and others^5,6,8,28–30^. We previously isolated *Lactobacillus johnsonii* strain N5 from stress-diarrhea-resistant animals and demonstrated its preventive effects in DSS-induced murine colitis, where it reduced weight loss, histopathological damage and inflammatory cytokine levels in the gut^31^. Furthermore, it rebalanced splenic Treg/Th17 responses by expanding the Treg population and reducing IL-17A production in Th17 cells^32^, supporting its potential to promote host homeostasis. However, key questions remain: can strain N5 serve as a viable treatment for established IBD, or is its efficacy limited to prophylaxis? What role does NET-mediated regulation play in its protective mechanisms?

Here, we investigate the effects of both prophylactic and therapeutic *L. johnsonii* N5 administration in experimental murine colitis. We show that peroral administration of N5 reduces gut microbiota dysbiosis, enhances colonic mucus production, and promotes epithelial proliferation, improving gut permeability and lowering pro-inflammatory cytokines production in colitis. Furthermore, N5 alleviated DSS-induced gut-liver inflammation and systemic consequences in mice, mechanistically linked to NET modulation. Our findings highlight neutrophil-driven NETosis as a novel therapeutic target and position *L. johnsonii* N5 as a multifaceted probiotic candidate for IBD and related inflammatory disorders.

## Results

### *Lactobacillus johnsonii* N5 protects against DSS-induced colitis in mice

**Fig. 1:**
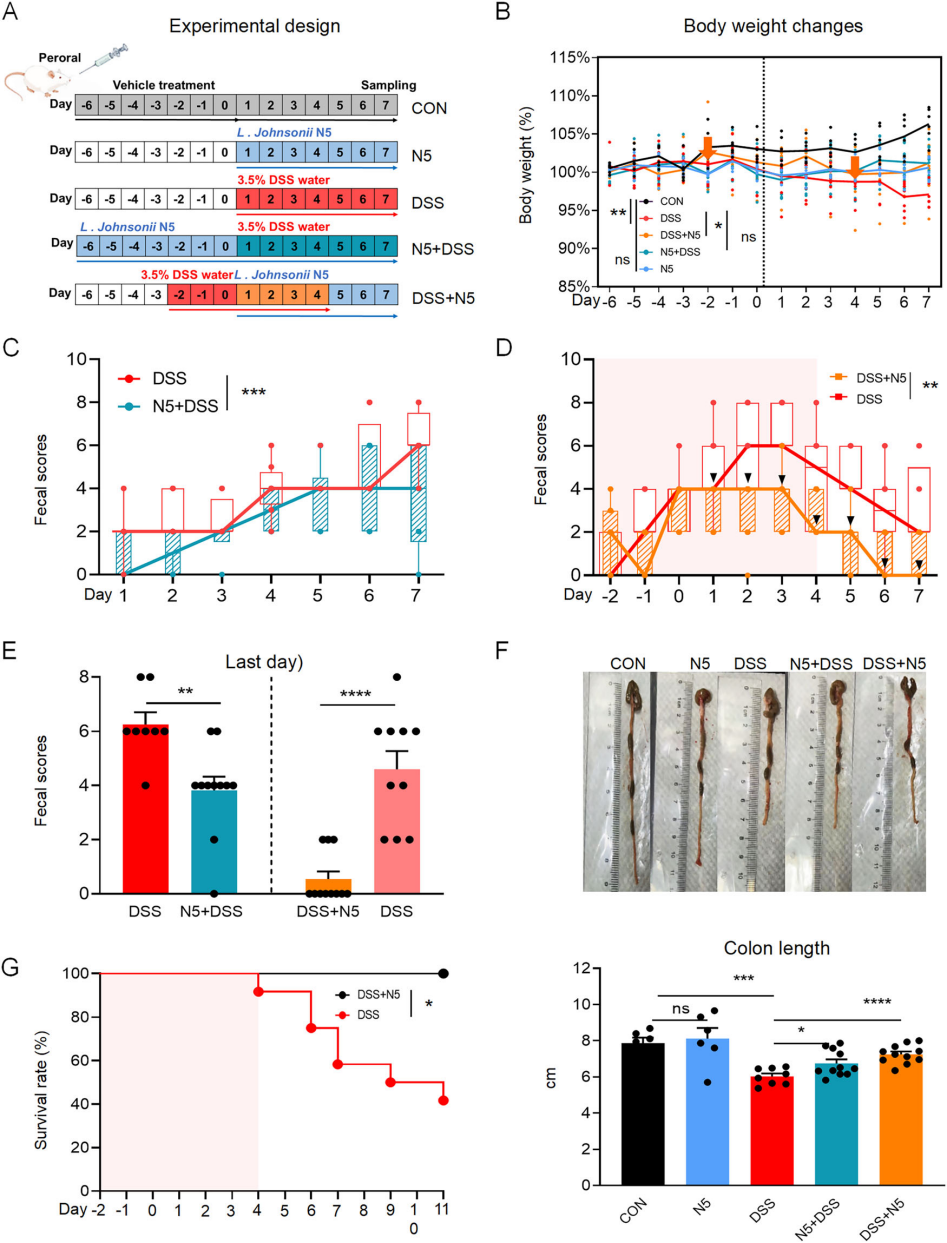
*Lactobacillus johnsonii* N5 protects against dextran sulphate sodium-induced colitis in mice. **A** Experimental design for *L. johnsonii* N5 administration (Created by Figdraw). BALB/c mice received 3.5% DSS in drinking water for 7 days to induce colitis. Treatment regimens: N5 group, daily oral gavage of *L. johnsonii* N5 (10^8^ CFU) for 7 days; N5 + DSS group, *L. johnsonii* N5 administered daily for 14 days, starting 7 days prior to DSS treatment; DSS + N5 group, therapeutic application of *L. johnsonii* N5 daily for 7 days, beginning on the 4th day of DSS treatment (clinical symptoms, e.g., soft stool, first observed at this timepoint). Whereas healthy mice from control group (CON) received vehicle treatment when appropriate. **B** Percentage change in body weight across groups. **C**–**E** Fecal scores in N5 + DSS and DSS + N5 groups compared to DSS controls. *Note*: Fecal scores for DSS mice in panels (**D**) and (**E**) were recorded on day 7 in a separate experiment, where DSS was withdrawn after 3 days to align with the DSS + N5 treatment timeline. Changes in body weight and fecal scores over time were analyzed and compared using the area under the curve (AUC). **F** Colon length (cm) measured post-treatment. **G** Survival rates (%) of DSS and DSS + N5 groups. Red shading indicates the duration of DSS treatment; data collection continued after treatment cessation (from day 7). Data are presented as mean ± SEM. *P < 0.05, **P < 0.01, ***P < 0.001, ****P < 0.0001 using two-tailed Student’s *t* test or ANOVA with Tukey’s post hoc test, ns not significant (*n* = 6–8 mice per group).

To evaluate the preventive and therapeutic effects of *Lactobacillus johnsonii* N5 in IBD, we induced colitis in 8–10-week-old male BALB/c mice using 3.5% DSS for 7 days (Fig. 1A). Mice were divided into five groups: healthy controls (CON); DSS-only controls; N5-only (daily oral gavage of *L. johnsonii* N5, 10^8^ CFU for 7 days); N5 + DSS (14-day pretreatment with *L. johnsonii* N5 starting 7 days before DSS); and DSS + N5 (therapeutic *L. johnsonii* N5 administered 10^8^ CFU for 7 days, from day 4 of DSS treatment onward, coinciding with symptom onset). Disease activity including body weight, stool consistency and the gross blood presence in feces was monitored daily as described^5,33^. DSS-treated mice exhibited significant weight loss compared to healthy controls (Fig. 1B, *P* < 0.01), which was mitigated in the DSS + N5 group (*P* < 0.05). However, prophylactic N5 + DSS pretreatment failed to prevent DSS-induced weight loss (*P* > 0.05). In contrast, fecal scores revealed robust protection in the N5 + DSS group, with significantly lower values throughout the experimental period (*P* < 0.001) and on day 7 compared to DSS controls (*P* < 0.01, Fig. 1C–E). Prophylactic N5 also attenuated DSS-induced colon shortening (Fig. 1F, *P* < 0.05). Therapeutic administration of N5 (DSS + N5 group) alleviated clinical symptoms, including rectal bleeding, diarrhea, and colon shortening (Fig. 1C–F, *P* < 0.05), and restored body weight (*P* < 0.05). Strikingly, DSS + N5 treatment conferred complete protection against DSS-induced mortality, which exceeded 60% in DSS-only mice by day 11 (7 days post-DSS withdrawal, Fig. 1G, *P* < 0.05). In addition, the effects of N5-only treatment were examined, and no difference was detected in body weight and colon length when compared to the control group (Fig. 1A, B, F, *P* > 0.05).

**Fig. 2:**
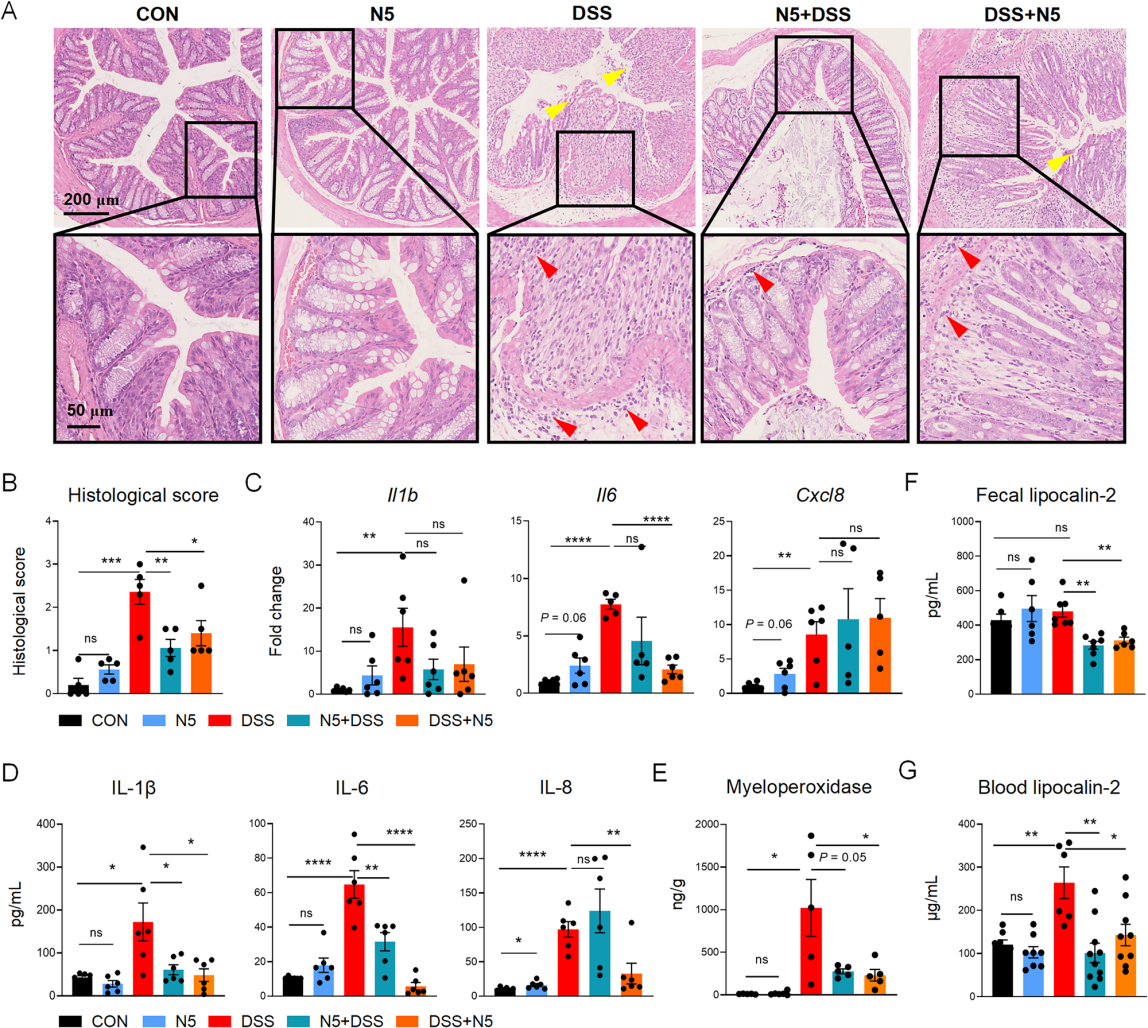
*Lactobacillus johnsonii* N5 protects against DSS-induced intestinal tissue damage and inflammation. **A** Representative images of H&E-stained colonic tissues from mice in the CON, N5, DSS, N5 + DSS, and DSS + N5 groups. Yellow arrows indicate epithelial damage at the mucosal surface; red arrows denote inflammatory cell infiltration in the submucosa and lamina propria. Scale bars = 200 μm or 50 μm (as indicated). **B** Histological scores of colon sections. **C** RT-qPCR analysis of pro-inflammatory cytokine gene expression (*Il1b*, *Il6*, *Cxcl8*) in colonic mucosa. Data were normalized to *Gapdh* expression. **D** Colonic concentrations (pg/mL) of IL-1β, IL-6, and IL-8. **E** Myeloperoxidase levels (ng/g tissue protein) in colon tissue. **F**, **G** Fecal lipocalin-2 concentrations (pg/mL) and blood lipocalin-2 levels (μg/L). Data are presented as mean ± SEM. *P < 0.05, **P < 0.01, ****P < 0.0001 using ANOVA with Tukey’s post hoc test; ns not significant (*n* = 5–10 mice per group).

Consistent with changes in clinical evaluations, histological analyses of distal colonic tissues exhibited epithelial damage (yellow arrows, Fig. 2A) and substantial immune cell infiltration (red arrows, Fig. 2A) in DSS-treated mice. In contrast, mice treated with N5—either prophylactically (N5 + DSS) or therapeutically (DSS + N5)—exhibited reduced histological signs of tissue damage and inflammation, with no significant differences observed between the N5-only group and healthy controls (Fig. 2A). Specifically, DSS treatment markedly increased histological score of distal colons compared to untreated controls (Fig. 2B, *P* < 0.001), while both N5 + DSS (*P* < 0.01) and DSS + N5 (*P* < 0.05) treatments significantly reduced these scores. No difference was observed between the N5-only and the control group (*P* > 0.05).

Pro-inflammatory cytokine levels in distal colonic tissues were quantified. Real time quantitative (RT-qPCR) analysis demonstrated that DSS treatment upregulated the gene expressions of *Il1b* (*P* < 0.01), *Il6* (*P* < 0.0001) and *Cxcl8* (encoding IL-8, *P* < 0.01) compared to the controls (Fig. 2C). Notably, *Il6* expression was significantly lower in DSS + N5 mice relative to the DSS group (Fig. 2C, *P* < 0.0001), though *Il1b* and *Cxcl8* levels remained unchanged (*P* > 0.05). Protein-level analysis mirrored these trends: DSS increased colonic IL-1β (*P* < *0.05*), IL-6 (*P* < 0.0001), and IL-8 (*P* < 0.0001) compared to controls (Fig. 2D). Prophylactic N5 + DSS treatment reduced IL-1β (*P* < 0.05) and IL-6 (*P* < 0.01), while therapeutic DSS + N5 administration suppressed all three cytokines (*P* < 0.05). Neutrophil infiltration, a hallmark of colitis^34^, was assessed via myeloperoxidase (MPO) activity^5,34^. DSS significantly increased MPO levels compared to controls (*P* < 0.05), whereas both N5 regimens suppressed this increase (Fig. 2E, *P* ≤ 0.05). Lipocalin-2 (neutrophil gelatinase-associated lipocalin, NGAL), a biomarker of IBD secreted by neutrophils to inhibit microbial growth^35,36^, was also evaluated. While DSS did not elevate fecal lipocalin-2 (Fig. 2F, *P* > *0.05*), N5-associated treatments reduced its levels post-DSS challenge (*P* < 0.01). Conversely, blood lipocalin-2 was elevated in DSS-treated mice (*P* < 0.01) and normalized by DSS + N5 (*P* < 0.05), but not by other treatments (*P* > 0.05). Critically, N5-only treatment caused no adverse effects: colon morphology (Fig. 2A, B), inflammatory protein levels (Fig. 2C–E), or blood lipocalin-2 (Fig. 2G) remained indistinguishable from controls (*P* > 0.05).

### *L. johnsonii* N5 protects against DSS-induced gut microbiota dysbiosis

**Fig. 3:**
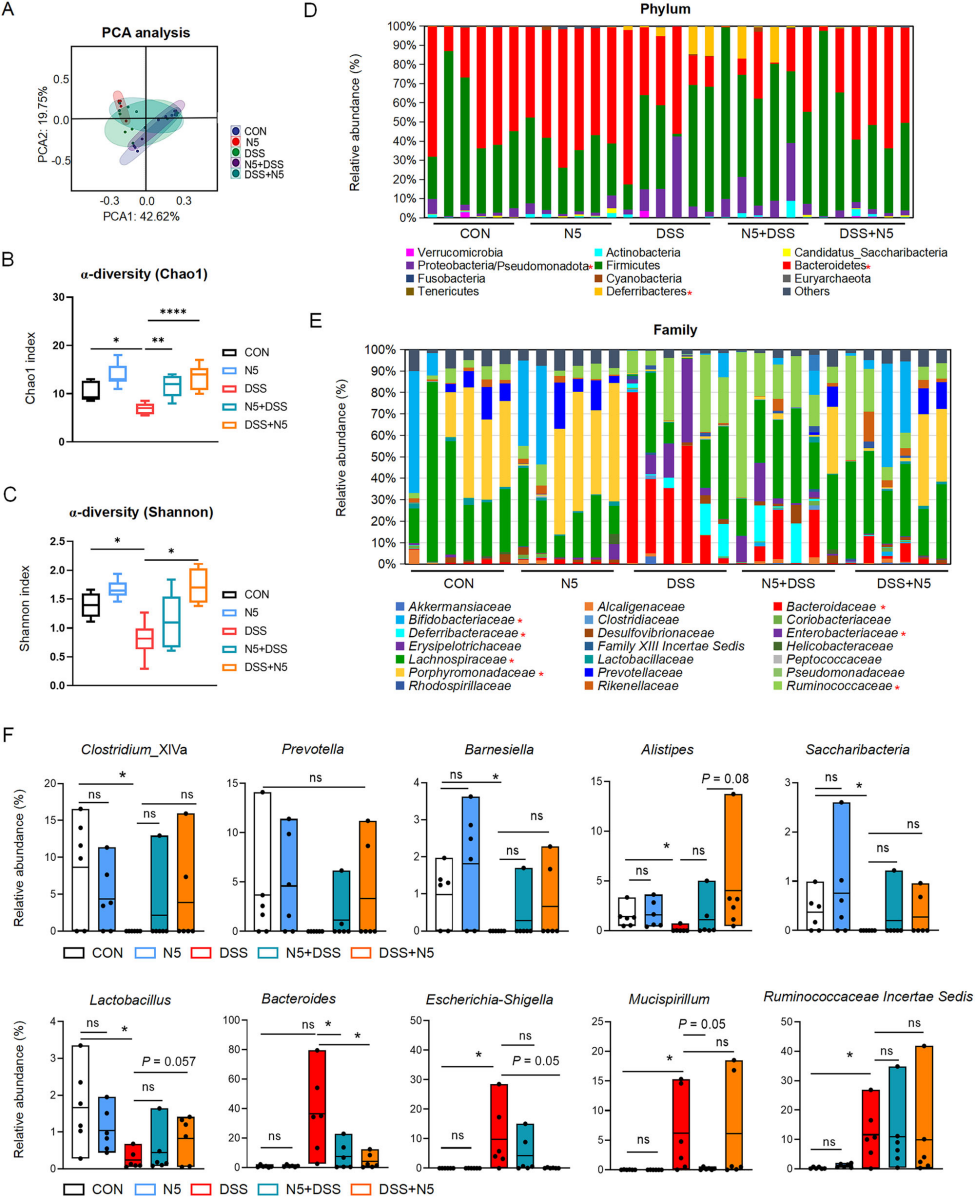
*Lactobacillus johnsonii* N5 protects against intestinal microbiota dysbiosis induced by DSS treatment. Fecal microbiota was examined by 16S rDNA sequencing. **A** Microbial community was assessed by Principal Component Analysis (PCA) and α-diversity was calculated as Chao1 (**B**) and Shannon index (**C**). Relative abundance of bacterial taxa at **D** phylum level and **E** family level. **F** Relative abundance of *Clostridium_*XlVa, *Prevotella*, *Barnesiella*, *Alistipes*, *Saccharibacteria, Lactobacillus*, *Bacteroides*, *Escherichia-Shigella*, *Mucispirillum* and *Ruminococcaceae Incertae*. Data are presented as mean ± SEM, *P < 0.05, **P < 0.01, ****P < 0.0001 (*P*-values were adjusted with false discovery rate control using the method of Benjamini and Hochberg) (*n* = 6 mice per group), ns not significant.

While our prior work established *L. johnsonii* N5 as a stress-resistant probiotic strain^31,32^, the role of gut microbiota in mediating its protective effects remained unclear and was elucidated here. First, the baseline composition of the fecal microbiota was determined prior to treatment initiation. Two samples per group (pooled by cage to account for co-housing effects) underwent 16S rRNA gene sequencing^37^. Principal component analysis (PCA) and α-diversity (Chao1 index) revealed no significant differences between groups at baseline (Supplementary Fig. S1A, B). Phylum and family level microbiota compositions exhibited expected inter-individual variability (Supplementary Fig. S1C, D). Consistent with previous studies^6,29,37^, DSS-induced colitis disrupted gut microbiota structure, evidenced by altered bacterial community clustering in PCA plots (weighted UniFrac distance; Fig. 3A) and reduced α-diversity (Chao1 and Shannon indices; Fig. 3B, C, *P* < 0.05). In contrast, N5 administration preserved α-diversity in DSS-treated mice (*P* < 0.05).

Further taxonomic analysis revealed DSS-driven shifts, including reduced Bacteroidetes, but increased Proteobacteria/Pseudomonadota and Deferribacteres at phylum level (Fig. 3D, *P* < 0.05). At family level, the relative abundance of *Bacteroidaceae*, *Enterobacteriaceae*, *Deferribacteraceae* and *Ruminococcaceae* was increased in DSS-treated mice compared to the control group (*P* < 0.05). Whereas significant reductions in the population of *Porphyromonadaceae*, *Lachnospiraceae* and *Bifidobacteriaceae* were detected (Fig. 3E, *P* < 0.05). Importantly, peroral administration of N5 reduced the DSS-induced gut microbiota dysbiosis. DSS + N5 treatment resulted in decreased abundances of Proteobacteria/Pseudomonadota and *Deferribacteres*, and increased abundances of *Lachnospiraceae*, *Porphyromonadaceae* and *Bifidobacteriaceae* (Fig. 3D, E, *P* < 0.05). Furthermore, the relative abundances of *Clostridium_*XlVa, *Barnesiella*, *Alistipes*, *Saccharibacteria* and *Lactobacillus* were decreased (*P* < 0.05), while those of *Escherichia-Shigella*, *Mucispirillum* and *Ruminococcaceae Incertae Sedis* were increased by DSS-induced colitis (Fig. 3F, *P* < 0.05). In contrast, the N5-associated treatments-maintained populations of *Lactobacillus*, *Bacteroides*, *Escherichia-Shigella* and *Mucispirillum* against DSS-induced dysbiosis (Fig. 3F, *P* < 0.05). N5-only treatment caused no significant microbiota alterations compared to healthy controls (Fig. 3A–F, *P* > 0.05).

### *L. johnsonii* N5 restores DSS-induced colonic barrier disruptions and transcriptome alterations

**Fig. 4:**
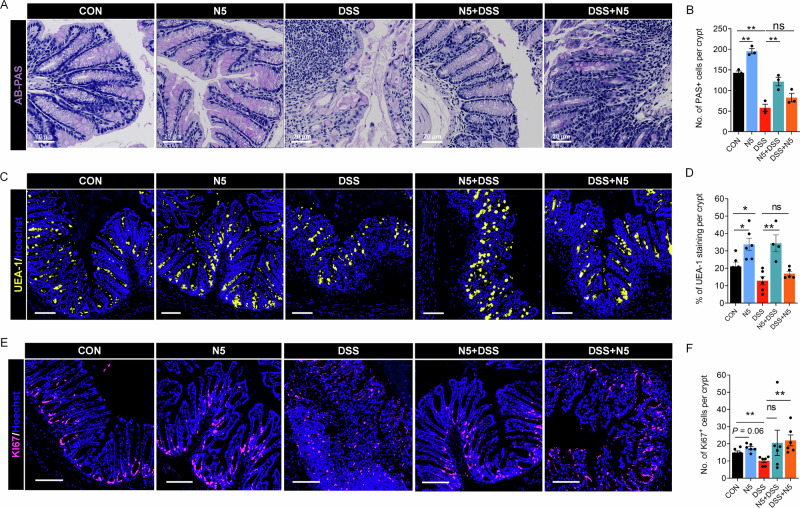
*Lactobacillus johnsonii* N5 restores DSS-induced intestinal barrier disruptions. **A** Representative images of distal colon sections stained for PAS^+^ goblet cells. **B** Enumeration of PAS^+^ goblet cells per crypt (each data point represents the mean of 10 crypts per mouse). **C** Representative images of distal colon sections stained for fucosylated mucin residues (Ulex Europaeus Agglutinin I [UEA-1] lectin, yellow) and nuclei (Hoechst, blue). **D** Quantification of UEA-1^+^ mucin area (% per crypt). **E** Representative images of distal colon sections stained for proliferating cells (Ki67 antibody, magenta) and nuclei (Hoechst, blue). **F** Number of Ki67+ proliferative cells per crypt. Scale bars = 100 μm (*n* = 6 mice per group, two slides analyzed per mouse). Data are presented as mean ± SEM, *P < 0.05, **P < 0.01 using ANOVA with Tukey’s post hoc test, ns not, significant.

The loss of mucin-producing goblet cells and the associated disruption of the mucus barrier, is a central pathological event in UC^38^. To assess intestinal barrier integrity and epithelial regeneration, we analyzed MUC glycosylation using staining with Alcian blue/periodic acid–Schiff (AB-PAS) and Ulex europaeus agglutinin I (UEA-1) lectin^39^, evaluated cell proliferation via Ki67 staining in the distal colon of DSS-treated mice with or without *L. johnsonii* N5 intervention (Fig. 4). DSS-induced colitis significantly reduced PAS^+^ goblet cell numbers (Fig. 4A, B, *P* < 0.01) and UEA-1^+^ mucin area (Fig. 4C, D, *P* < 0.05) compared to healthy controls. Prophylactic N5 pretreatment (N5 + DSS) restored PAS^+^ cell counts (*P* < 0.01) and UEA-1^+^ mucin (*P* < 0.01), whereas therapeutic N5 administration (DSS + N5) showed no significant improvement (*P* > 0.05). DSS also reduced epithelial proliferation, marked by fewer Ki67^+^ cells in the distal colon (Fig. 4E, F, *P* < 0.01). Therapeutic N5 (DSS + N5) partially rescued this effect, increasing Ki67^+^ cells compared to DSS alone (*P* < 0.01), but prophylactic N5 (N5 + DSS) had no impact (*P* > 0.05). Notably, N5-only treatment enhanced mucus barrier components, increasing PAS^+^ goblet cells (*P* < 0.01) and UEA-1^+^ mucin (*P* < 0.05) relative to controls, with a trend toward elevated Ki67^+^ cell numbers (*P* = 0.06).

**Fig. 5:**
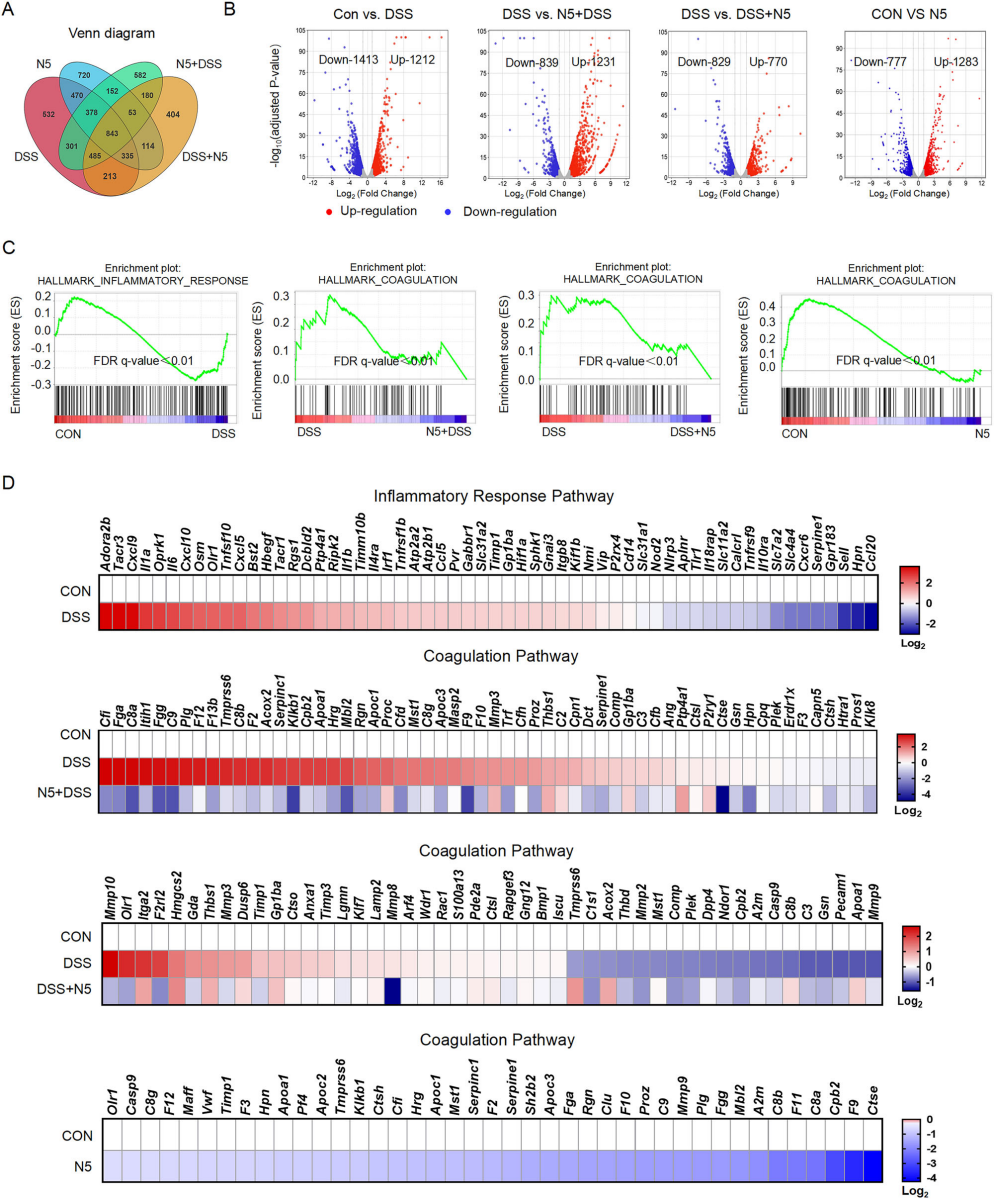
*Lactobacillus johnsonii* N5 modulates colonic transcriptome program alteration induced by DSS treatment in mice. **A** A Venn diagram of the number of genes significantly differentially expressed (Log_2_ expression, fold change > 1) in the colon of mice between DSS vs. CON, N5 vs. CON, N5 + DSS vs. DSS and DSS + N5 vs. DSS, determined by RNA-seq analysis. **B** Volcano plot visualization of the differentially expressed genes between DSS vs. CON, N5 + DSS vs. DSS, DSS + N5 vs. DSS and N5 vs. CON. **C**, **D** GSEA plots and Heatmaps depicting the enrichment of genes up- or down-regulated in the inflammatory response pathway and coagulation pathway. Hypergeometric test and Benjamini–Hochberg *p*-value correction (FDR false-discovery rate) was applied.

To delineate the distinct protective effects of prophylactic versus therapeutic *L. johnsonii* N5 administration in DSS-induced intestinal inflammation, dysbiosis, and barrier dysfunction, we performed RNA-Seq analysis on distal colonic tissues. Transcriptomic profiling (|Log2 (fold change)|>1) revealed divergent gene expression patterns between DSS-treated mice and controls, N5 and controls, N5 + DSS and DSS, and DSS + N5 and DSS (Fig. 5A). Volcano plots highlighted 1212 upregulated and 1413 downregulated genes in DSS vs. controls, 1231 upregulated and 839 downregulated genes in N5 + DSS vs. DSS, 770 upregulated and 829 downregulated genes in DSS + N5 vs. DSS, and 1283 upregulated and 777 downregulated genes in N5-only vs. controls (Fig. 5B). Gene Set Enrichment Analysis (GSEA) demonstrated that DSS-treated mice exhibited significant enrichment in the ‘hallmark inflammatory response’ pathway compared to controls. Conversely, both N5 + DSS and DSS + N5 treatments suppressed the DSS-induced upregulation of the ‘hallmark coagulation’ pathway (Fig. 5C, FDR *q*-value < 0.01), which is mechanistically linked to thrombosis^40^. This effect was also observed when comparing N5-only treatment to controls (Fig. 5C, FDR *q*-value < 0.01). In consistency, heatmap analysis further identified DSS-driven upregulation of ‘inflammatory response pathway’ (Fig. 5D, upper panel), coagulation associated genes of *Cfi*, *Fga*, *C8a*, *Itih1*, *Fgg*, *C9*, *Plg*, *F12*, *F13b* and *Acox2* (Fig. 5D, middle panel), as well as *Mmp10*, *Olr1*, *Itga2*, *F2rl2*, *Hmgcs2*, *Gda*, *Thbs1*, *Mmp3*, *Dusp6*, and *Timp1* (Fig. 5D, lower panel), which were attenuated by N5 + DSS and DSS + N5 treatments, respectively. In addition, it was also showed that N5-only treatment resulted in downregulation of *Olr1*, *Casp9*, *C8g*, *F12*, *Maff*, *Vwf*, *Timp1*, *F3*, *Hpn*, *Apoa1*, *Pf4*, *Apoc2*, *Tmprss6*, *Klkb1*, *Ctsh*, *Cfi*, *Hrg*, *Apoc1*, *Mst1*, *Serpinc1*, *F2*, *Serpine1*, *Sh2b2*, *Apoc3*, *Fga*, *Rgn*, *Clu*, *F10*, *Proz*, *C9*, *Mmp9*, *Plg*, *Fgg*, *Mbl2*, *A2m*, *C8b*, *F11*, *C8a*, *Cpb2*, *F9*, and *Ctse* genes of ‘coagulation pathway’, compared to controls (Fig. 5D, bottom panel).

### *L. johnsonii* N5 protects against DSS-induced bacterial translocation and associated liver injury

**Fig. 6:**
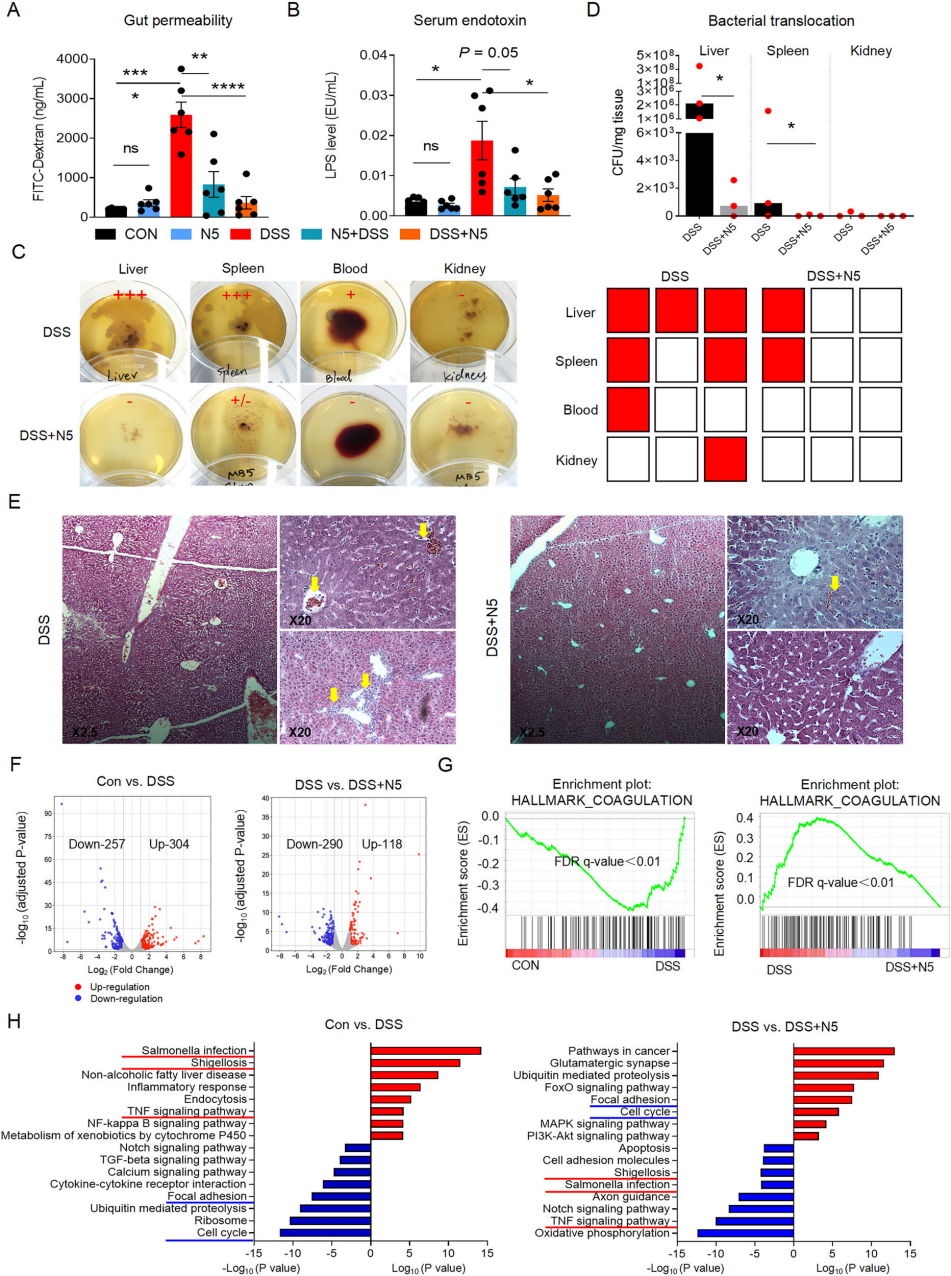
*Lactobacillus johnsonii* N5 protects against DSS-induced bacterial translocation and the associated liver injury. **A** Intestinal permeability determined using fluorescein isothiocyanate-dextran (FITC, 4 K DA) oral gavage assay (ng/mL). **B** Serum endotoxin (LPS levels) (*n* = 6 mice per group). **C** Representative images of bacterial translocation assessed in the liver, spleen, blood and kidney (**D**) and quantified as colony forming units (CFU) per mg tissue of liver, spleen and kidney (*n* = 3 mice per group, repeated at least twice). **E** Representative images of liver sections stained with H&E from DSS and DSS + N5 groups at 2.5× and 20× magnification, respectively. The yellow arrows show mononuclear cell infiltration at the portal area and blood cell accumulation. **F** Volcano plot visualization of the differentially expressed genes between DSS vs. CON and DSS + N5 vs. DSS. **G** GSEA plots depicting the enrichment of genes altered in coagulation pathway (FDR false-discovery rate). **H** The enrichment pathways analyzed by the KEGG between DSS vs. CON and DSS vs. DSS + N5. Data are presented as mean ± SEM. *P < 0.05, **P < 0.01, ****P < 0.0001 using ANOVA with Tukey’s post hoc test, ns not significant.

To further assess intestinal barrier function, gut permeability assays were conducted by orally administering fluorescein isothiocyanate conjugated dextran (FITC-dextran) to mice as previously described^8^. Results revealed significantly elevated serum FITC-dextran levels in DSS-treated mice compared to healthy controls (Fig. 6A, *P* < 0.0001), which were attenuated by both prophylactic (N5 + DSS, *P* < 0.01) and therapeutic (DSS + N5, *P* < 0.0001) N5 administration. DSS also increased systemic endotoxemia, evidenced by elevated blood LPS levels (*P* < 0.05), while N5-associated treatments (N5 + DSS, *P* = 0.05; and DSS + N5, *P* < 0.05) reduced circulating endotoxin (Fig. 6B). To evaluate bacterial translocation, viable bacteria were cultured from tissues and blood. DSS-treated mice exhibited higher bacterial loads in the liver and spleen compared to the DSS + N5 group (*P* < 0.05), with no detectable bacteria in kidneys or blood of DSS + N5 mice (Fig. 6C, D).

Histopathological analysis of liver tissues from DSS-treated mice revealed mononuclear cell infiltration (yellow arrows, Fig. 6E), disorganized hepatic cords, and vascular occlusions, all of which were mitigated by DSS + N5 treatment. Furthermore, RNA-Seq of liver tissues identified 304 upregulated and 257 downregulated genes in DSS vs. controls, and 118 upregulated and 290 downregulated genes in DSS + N5 vs. DSS (Fig. 6F). GSEA highlighted DSS-driven upregulation of the “hallmark coagulation” pathway (indicative of tissue injury), which was suppressed by DSS + N5 (Fig. 6G, FDR *q* < 0.01). Gene Ontology (GO) enrichment analysis further showed that DSS upregulated pathways linked to infection (*Salmonella* infection, shigellosis) and inflammation (TNF signaling, non-alcoholic fatty liver disease), while DSS + N5 downregulated these pathways and inhibited oxidative phosphorylation, Notch signaling, and apoptosis (Fig. 6H). Notably, DSS + N5 restored focal adhesion and cell cycle pathways, suggesting enhanced tissue repair^41^.

**Fig. 7:**
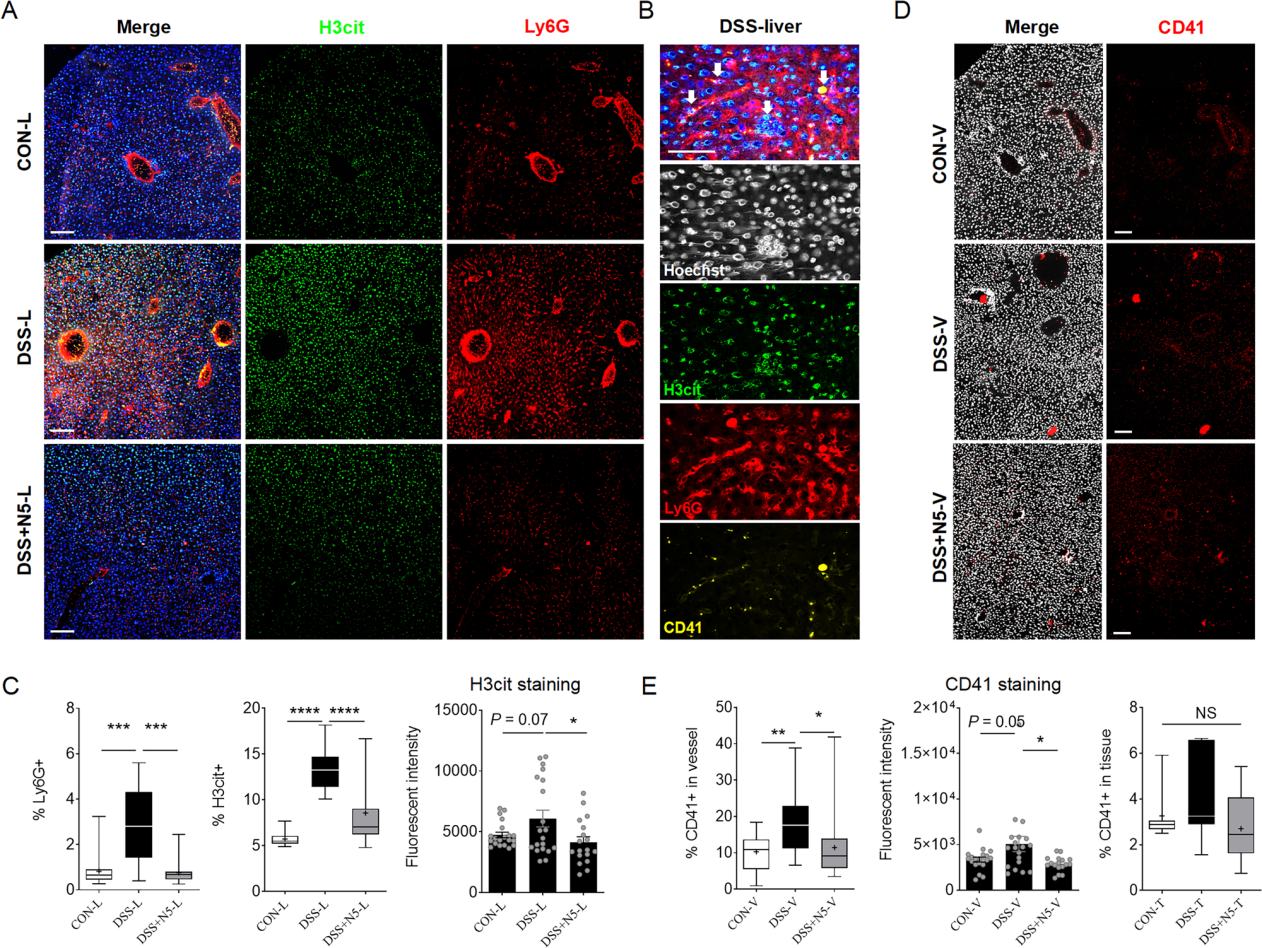
*Lactobacillus johnsonii* N5 reduces neutrophil-mediated liver disruption in DSS-induced colitis. **A** Representative images of liver tissue sections stained with anti-H3cit (green), anti-Ly6G (red) and anti-CD41 (yellow) from various groups. The nuclei were stained with Hoechst (blue), scale bars = 100 μm. **B** Representative images of liver tissue from DSS-treated mice showing spots identified as NETs with H3cit^+^ neutrophil infiltration and nuclear damage, scale bars = 50 μm. **C** Analysis and quantification of the percentage of Ly6G^+^ and H3cit^+^ area in liver tissues, and H3cit staining levels (Fluorescent intensity). **D** Representative images of staining for anti- CD41 antibody (red) in vessel and in liver tissues, scale bars = 100 μm. **E** Analysis and quantification of the percentage of CD41^+^ area in vessels and in tissues, and CD41 staining levels (Fluorescent intensity). Scale bars = 100 μm (*n* = 6 mice per group, two slides per mouse). Data are presented as mean ± SEM, *P < 0.05, **P < 0.01, ***P < 0.001 and ****P < 0.0001 using ANOVA with Tukey’s post hoc test, ns not significant.

Utilizing immunofluorescent staining in combination with confocal microscopy, our study demonstrated that inflammation induced by DSS was associated with significant neutrophil infiltration in the liver. The analysis indicated a notably elevated percentage of Ly6G^+^ neutrophils in the DSS-treated group when compared to the healthy control group, as well as DSS + N5 treatment group (Fig. 7A–C, *P* < 0.001). Further analysis revealed the presence of NETs in the inflicted liver tissues, characterized by Ly6G and H3cit (a biomarker for early NETosis detection) positivity, as well as neutrophil-derived net-like structures (Fig. 7B). These structures contained granule proteins and chromatin-histone complexes, consistent with established NET composition^13^. Therapeutic N5 administration (DSS + N5) reduced H3cit^+^ cell accumulation (Fig. 7C, *P* < 0.0001) and showed a trend toward lower H3cit fluorescence intensity (*P* = 0.07), suggesting suppression of NETosis. In addition, CD41 staining for thrombocytes in the liver (Fig. 7D, E), showed a significant increase in the hepatic vessels of the DSS-treated mice (*P* < 0.05), indicative of blood occlusions, whereas this was reduced by DSS + N5 treatment (*P* < 0.05), while CD41 distribution in parenchymal tissues remained unchanged (*P* > 0.05).

### DNase I recapitulates the protective effect of *Lactobacillus johnsonii* N5 involving NETs-mediated gut-liver inflammation in experimental colitis

**Fig. 8:**
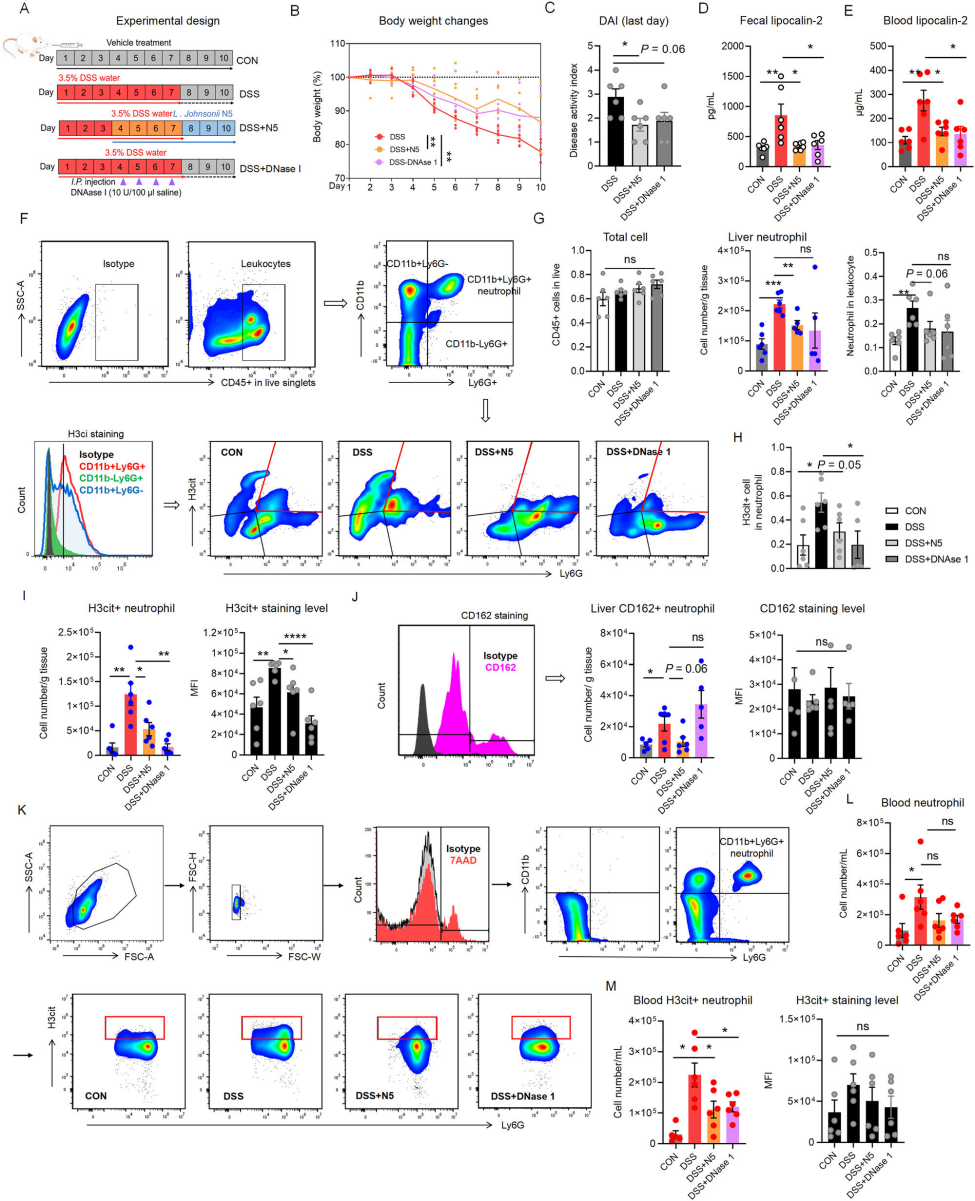
*Lactobacillus johnsonii* N5 provides protection against neutrophil extracellular traps (NETs)-mediated liver disruption in DSS-induced colitis. **A** Experimental design for evaluating therapeutic effects of *L. johnsonii* N5 and DNase I (Created by Figdraw). **B** Body weight changes (%) across groups and **C** disease activity index (DAI) at endpoint (day 10). **D** Fecal lipocalin-2 (pg/mL) and **E** serum lipocalin-2 (μg/L) levels. **F** Flow cytometry analysis of hepatic neutrophils (gated as live CD45^+^CD11b^+^Ly6G^+^) and a histogram of H3cit staining in these cells. **G** The percentage of CD45^+^ leukocytes in total, the percentage of neutrophil in leukocytes and the number of neutrophils per g liver tissue. **H**, **I** Flow cytometry quantification of the frequency and the absolute number of H3cit^+^ neutrophils per g liver tissue and H3cit staining levels (Median fluorescent intensity, MFI) in these cells. **J** Quantification of the number of CD162^+^ neutrophils and CD162 staining levels (MFI) in these cells. **K** Flow cytometry analysis of blood neutrophils (live CD11b^+^Ly6G^+^) and the H3cit^+^ subset. Quantification of the number of neutrophils per mL (**L**), the H3cit^+^ subset and H3cit MFI in these cells in blood (**M**). Data are presented as mean ± SEM, *P < 0.05, **P < 0.01, ***P < 0.001 and ****P < 0.0001 using ANOVA with Tukey’s post hoc test, ns not significant.

To further explore the involvement of neutrophils and NETs in DSS-induced colitis, we compared the therapeutic effects of DNase I (a NET-degrading agent^42^) and *L. johnsonii* N5. Both treatments mitigated DSS-induced body weight loss (Fig. 8A, B, *P* < 0.01). DNase I exhibited a tendency to reduce colitis symptoms (DAI score, *P* = 0.06), while N5 alleviated disease severity (*P* < 0.05; Fig. 8C). DNase I also mirrored N5’s anti-inflammatory effects, normalizing fecal and serum lipocalin-2 levels to those of healthy controls and DSS + N5 mice (Fig. 8D, E, *P* < 0.05).

Flow cytometry analysis of hepatic leukocytes revealed no differences in total CD45^+^ cell counts across groups (Fig. 8F, G, *P *> 0.05). However, N5 significantly reduced DSS-driven hepatic neutrophil infiltration (CD11b^+^Ly6G^+^ cells, *P* < 0.01) and their proportion among leukocytes (*P* = 0.06), restoring levels to near-control values (*P* < 0.01). DNase I had no effect on neutrophil counts (*P* > 0.05). DSS increased hepatic H3cit^+^ neutrophils (Fig. 8F, I, *P* < 0.05), which were reduced by DNase I (*P* < 0.05) and N5 (*P* < 0.05). N5 also lowered H3cit median fluorescence intensity (MFI) in neutrophils (*P* < 0.05), suggesting suppressed NETosis. While N5 partially reversed DSS-induced CD162^+^ neutrophil expansion (Fig. 8J, *P* = 0.06), DNase I showed no effect (*P* > 0.05). In blood, DSS increased circulating CD11b^+^Ly6G^+^ neutrophils and H3cit^+^ subsets (Fig. 8K–M, *P* < 0.05) without altering H3cit MFI. Both DNase I and N5 reduced circulating H3cit^+^ neutrophil populations (*P* < 0.05).

**Fig. 9:**
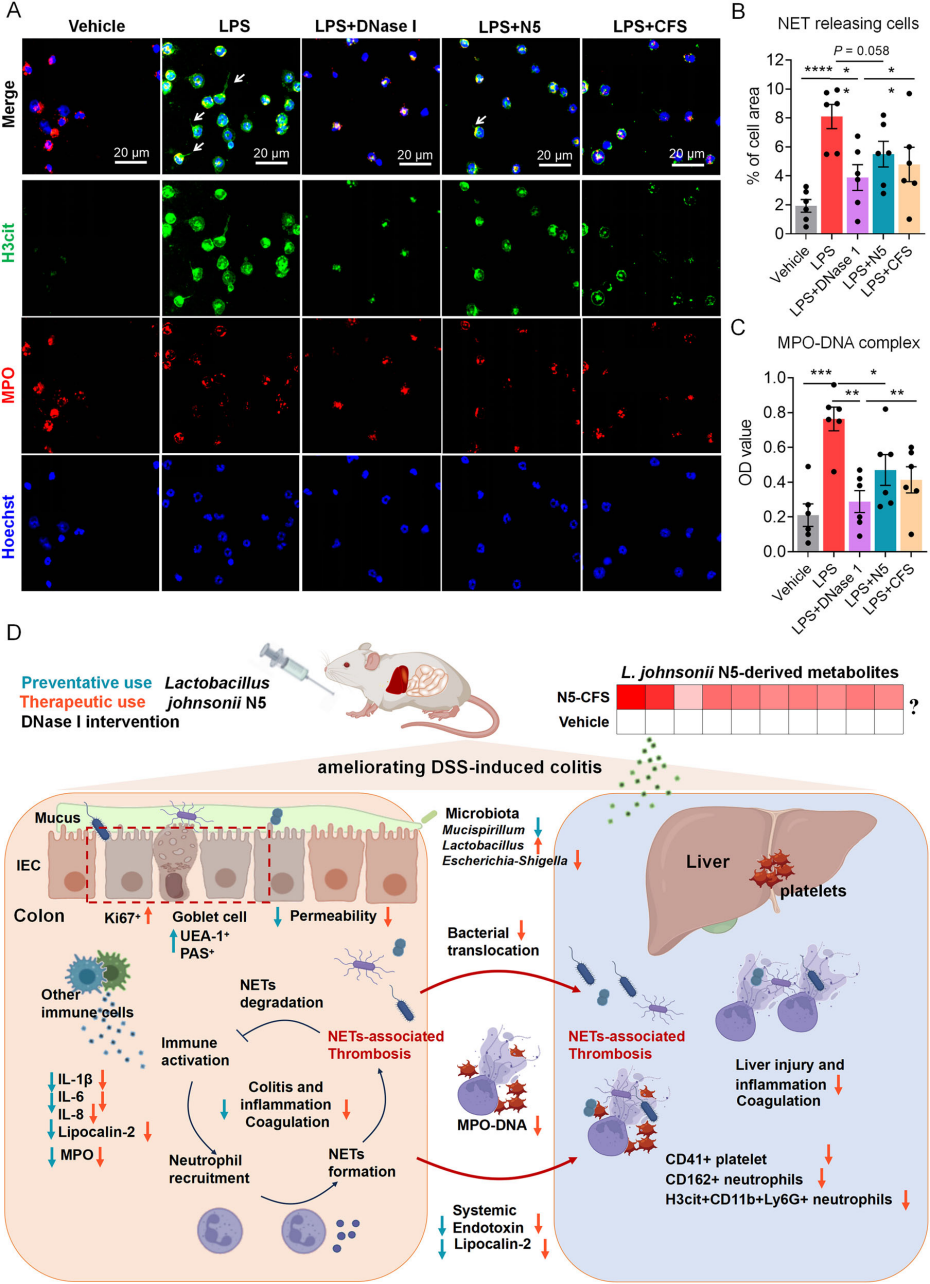
*Lactobacillus johnsonii* N5 suppresses LPS-induced NET formation and attenuates DSS-induced colitis: mechanisms and therapeutic implications. **A**, **B** Microscopy images of NET formation in bone marrow-derived neutrophils (BMDNs), incubated with LPS (5 μg/mL) stimulation, or in combination of DNase I (200 U/mL), or *L. johnsonii* N5 (1 × 10^8^ CFU/mL), or CFS (N5 equivalent). NETs were staining for DNA (Hoechst, blue), H3-cit (green) and MPO (red) and NET releasing cells were quantified by measuring the area of MPO+/histone H3+ extracellular DNA structures in five random fields per sample using ImageJ software, normalized to 100 cells (**B**). **C** BMDN levels of NET-DNA were detected using an MPO-DNA ELISA kit and the results were expressed as mean OD values. Data are presented as mean ± SEM, from at least six indipendnt experiemnts. *P < 0.05, **P < 0.01 and ***P < 0.001 using ANOVA with Tukey’s post hoc test, ns not significant. **D** Schematic illustration of the preventative and therapeutic effects of *L. johnsonii* N5 regulation in DSS*-*induced colitis in mice. CFS cell free supernatant, DSS dextran sodium sulfate, IL-6 interleukine-6, IEC Intestinal epithelial cell, MPO Myeloperoxidase, NETs neutrophil extracellular traps, UEA-1 Ulex europaeus agglutinin I.

To assess direct effects on NET formation, BMDNs from tibias and femurs of mice were treated in vitro with LPS (5 μg/ml) alone or in combination with DNase I (200 U/mL), *L. johnsonii* N5 (10^8^ CFU/mL), or N5 cell-free supernatant (CFS). LPS significantly increased NETosis, measured as the percentage of cells releasing NETs (determined by co-localization of H3cit and MPO), compared to vehicle controls (Fig. 9A, B, *P* < 0.01), while DNase I (*P* < 0.01) and CFS (*P* < 0.01) suppressed this effect. LPS + N5 showed a trend toward reducing NET release (*P* = 0.05). ELISA confirmed LPS-induced MPO-DNA complex production (Fig. 9C, *P* < 0.01), which was attenuated by DNase I (*P* < 0.001), N5 (*P* < 0.05), and CFS (*P* < 0.01). These results suggest that N5 alleviated gut-liver inflammation partially via NET suppression, with metabolites in CFS contributing to this effect (Fig. 9D). In addition, untargeted metabolomics of N5 CFS identified several metabolites, including 3-phenyllactic acid, N-acetylornithine, and DL-lactic acid etc. (Supplementary Data 1). While these metabolites may mediate NET inhibition, their individual roles require further validation.

## Discussion

Ulcerative colitis (UC) is a multifactorial disease requiring therapeutic strategies that address both mucosal healing and systemic inflammation^1,32^. Our study demonstrates that *Lactobacillus johnsonii* N5 exerts dual protective roles in experimental colitis, mediated in part by its metabolites. Prophylactically, N5 preserves gut microbiota diversity (e.g., preventing overgrowth of mucus-dwelling *Mucispirillum*), enhances colonic mucus secretion, reduces pro-inflammatory cytokines (IL-1β, IL-6), and reinforces mucosal barrier integrity. Therapeutically, N5 targets neutrophil-mediated gut-liver inflammation by suppressing NETosis and downstream coagulation pathways, thereby limiting bacterial translocation (e.g., *Escherichia-Shigella*), reducing thrombotic tendencies, and curtailing neutrophil recruitment and epithelial damage. Concurrently, N5 accelerates mucosal repair through enhanced epithelial proliferation. These findings position N5 as a multifaceted probiotic candidate with translational potential for IBD.

The prophylactic efficacy of N5 aligns with established probiotic mechanisms, including mucus layer preservation, *Lactobacillus* population maintenance^7,29,43,44^, and suppression of pathobionts during inflammatory cascades, thereby preventing the onset of IBD^5,32,45,46^. Complementing these findings, our previous work established that N5 downregulates IL-17 and TNF signaling while modulating HIF-1 and oxidative phosphorylation pathways in the spleen during DSS challenge. This suppresses splenic Th17 cell expansion and IL-17A production, restores Treg function, and ameliorates functional hyposplenism—an established extraintestinal manifestation of IBD^32^. Building on this, the current study demonstrates that N5 administration also prevented the elevation of *Mucispirillum*, a genus known to colonize the mucus layer^29^ and trigger spontaneous colitis in mice lacking NOD2 and CYBB (*Nod2*^*–/–*^*Cybb*^*–/–*^), while impairing neutrophil recruitment and antimicrobial activities^47^. Furthermore, N5 treatment ameliorated colitis by reinforcing mucus barrier integrity via promotion of PAS^+^ goblet cells and UEA-1^+^ mucin^39,46^. These effects synergistically limit direct pathogen-epithelial contact, thereby decreasing local inflammation (including pro-inflammatory cytokine production of IL-1β and IL-6)—a critical step in colitis initiation^46^.

Notably, previous studies have demonstrated that goblet cell depletion in colitis is closely linked to IL-8-induced neutrophil recruitment and the release of neutrophil-derived secretagogues^15,34^. Our study revealed DSS-induced neutrophil infiltration in colonic tissue, correlating with increased intestinal MPO activity, IL-8 production, and elevated blood lipocalin-2 levels, indicating neutrophil-driven local and systemic inflammation^35,36^. Consistent with this, we observed increased serum neutrophils and H3cit^+^CD11b^+^Ly6G^+^ cells in DSS-treated mice. NETs in the gut and/or blood have been implicated in IBD pathogenesis^21,22,48,49^, and murine colitis models recapitulate NETs formation in inflamed mucosa, characterized by DNA backbones decorated with neutrophil elastase, MPO, and H3cit^19,20^. Moreover, our transcriptomic analysis revealed DSS-induced enrichment of coagulation pathways in the colon, suggesting a pro-inflammatory hypercoagulable state. This aligns with recent work by Zhang et al., who demonstrated that inflammation-driven epithelial exosome secretion promotes thrombus formation in DSS-treated mice via NETs^20^. Notably, current IBD therapies, including biologics, fail in 30–40% of patients, often those with neutrophilic inflammation^3^.

Therapeutically, N5 diverges from conventional probiotics by uniquely targeting NETosis and neutrophil-mediated gut-liver inflammation, positioning it as a complementary therapy for biologic non-responders. Our data reveal that N5 administration reduces *Escherichia-Shigella* abundance, a pathobiont known to exacerbate inflammation through LPS and biofilm formation^24^, thereby limiting bacterial translocation to the liver. N5 also suppresses hepatic H3cit^+^ neutrophils and circulating NET-DNA complexes, and CD41^+^ platelet aggregation linked to portal vein occlusions, mirroring DNase I’s effects. While *Lactobacillus* species, including N5, are typically DNase-negative^50,51^, our in vitro experiments demonstrate that N5-derived metabolites directly suppress LPS-induced NETosis in BMDNs. Notably, untargeted metabolomics identified 3-phenyllactic acid in N5’s CFS, a compound with known antioxidant and anti-inflammatory properties which may inhibit NETosis by scavenging ROS or modulating neutrophil signaling pathways (*e.g*., NF-κB^52^). This mechanism aligns with LGG, which suppresses NETosis via ROS dampening^26^. However, unlike DNase I^18^ which solely degrades existing NETs, N5 combines metabolite-driven NET inhibition with microbiota restoration, offering a dual therapeutic advantage. These findings extend prior work demonstrating that probiotics ameliorate inflammation through diverse mechanisms, including direct suppression of NET-inducing pathogens (*E. coli*, *S. aureus*, *C. rodentium*) and inhibition of their mucosal adherence^24,25,53^. For instance, *Lactiplantibacillus plantarum*-derived 3-phenyllactic acid enhances stress resilience and energy metabolism in *Caenorhabditis elegans* via SKN-1/ATFS-1, a pathway homologous to human Nrf1/2 antioxidant regulators^54^, highlighting conserved antioxidant pathways. Similarly, tryptophan-metabolizing *Lactobacillus* activate aryl hydrocarbon receptor (AHR) signaling, modulating T cell responses to attenuate IBD^7,55^, further underscoring the multi-targeted anti-inflammatory potential of probiotics.

Nevertheless, several limitations in our study warrant consideration. First, reliance on the DSS murine model which primarily reflects acute epithelial injury, limits generalizability to chronic, immune-mediated human IBD. Complementary approaches, such as IL-10-deficient models, are needed to validate and extend these findings. Second, while metabolites like 3-phenyllactic acid were identified, their direct role in NET suppression requires validation via purified compound testing. Third, translating N5’s benefits to humans requires rigorous evaluation of dosage, viability, and safety, as well as synergy testing with anti-TNF biologics. Finally, the gut-liver axis mechanisms observed here merit deeper exploration in chronic models or patient-derived samples to establish clinical relevance.

In conclusion, our study elucidates *L. johnsonii* N5’s dual role in colitis management: prevention via barrier enhancement and treatment through NET suppression. By bridging gut-liver inflammation and microbial dysbiosis, these findings expand the paradigm of probiotic mechanisms and highlight neutrophils as therapeutic targets. While clinical translation requires further optimization, N5 represents a promising candidate for resolving unmet needs in IBD therapy, particularly in patients with neutrophil-predominant inflammation.

## Material and methods

### Animals

All animal procedures and experiments in this study were approved by The Animal Care and Use Committee of Yangzhou University [SYXK (SU) 2021-0026]. We have complied with the ARRIVE guidelines and all relevant ethical regulations for animal use. Wild-type male BALB/c mice were obtained from the Jiangsu Laboratory Animal Science Center and housed under specific pathogen-free conditions (22 °C, 50% humidity, 12/12-h light/dark cycle) with free access to water and standard chow. After one-week acclimation, mice (8–10 weeks old) were randomized into five groups: Healthy controls (CON), received vehicle treatment when appropriate; DSS controls, administered 3.5% DSS in drinking water for 7 days. N5 group, daily oral gavage of *L. johnsonii* N5 (10^8^ CFU/day) for 7 days; N5 + DSS group, pretreated with *L. johnsonii* N5 (10^8^ CFU/day) for 14 days (7 days pre-DSS + 7 days concurrent with DSS); and DSS + N5 group, therapeutic *L. johnsonii* N5 (10^8^ CFU/day) administered from day 4 of DSS treatment for 7 days.

In particular, colitis was induced via 3.5% (w/v) DSS (MW 36,000–50,000, Yeasen, Shanghai, China) in drinking water for 7 days. Disease activity (weight loss, stool consistency, fecal blood) was scored daily as described^5^. The assessment includes evaluations of weight loss, stool consistency was graded as normal (0), loose (2), or diarrhea (4); fecal blood was assessed using hemoccult tests (0: negative; 2: trace; 4: gross bleeding). *L. johnsonii* N5 was freshly suspended in PBS and administered orally (10^8^ CFU/mouse/day). For the DSS + N5 group, treatment began on day 4 of DSS exposure, coinciding with symptom onset (soft stool). Notably, separate animal experiments were carried out for fecal scoring regarding DSS + N5 and their corresponding DSS groups, where data were recorded until day 7 (3 days post-DSS withdrawal) to align treatment timelines. While survival rates were monitored for 7 days during DSS treatment and 7 days post-withdrawal.

### Probiotic cultures, cell-free supernatant preparation and DNase I treatment

*Lactobacillus johnsonii* N5 sourced from our laboratory^31^, deposited at the China Center for Type Culture Collection (CCTCC, NO:M2023104), was cultured in MRS broth (Oxoid) at 37 °C for 20 h under anaerobic conditions. Bacteria were harvested at the early stationary phase by centrifugation (5000 rpm for 10 min), washed twice with PBS, and concentrated 100-fold in a cryoprotective solution (0.82 g K_2_HPO_4_, 0.18 g KH_2_PO_4_, 0.59 g sodium citrate, 0.25 g MgSO_4_ × 7 H_2_O, and 172 mL glycerol (87%) diluted to 1000 mL with dH_2_O) and stored at −80 °C.

Cell-free supernatant (CFS) was prepared by centrifuging *L. johnsonii* N5 cultures (5000 rpm for 10 min), followed by sterile filtration of the supernatant through 0.22 μm membranes (Millipore). Filtered CFS was flash-frozen in liquid nitrogen and stored at −80 °C. Three independent biological replicates of CFS were prepared for downstream experiments. For co-culture experiment with isolated neutrophil, the concentration of CSF was modified to achieve an OD_595 nm_ value of ~1.0, indicating a viable bacterial population of 1 × 10^8^ CFU/mL and served as the working solution.

DNase I was used therapeutically to study the involvement of neutrophils and NETs in DSS-induced colitis. Separate sets of animal experiments were conducted, where mice (8–10 weeks old) were randomized into four groups (Fig. 8A): Healthy controls (CON), received vehicle treatment when appropriate; DSS controls, administered 3.5% DSS in drinking water for 7 days, followed by 3 days of DSS withdrawal; DNase I + DSS: treated with DNase I (10 U in 100 μL sterile saline, Thermo Fisher Scientific, Shanghai, China) via daily *i.p*. injection from day 4 of DSS treatment (symptom onset) for 4 days. Mice were sacrificed 3 days post-treatment. DSS + N5: received therapeutic *L. johnsonii* N5 (10^8^ CFU/day orally) from day 4 of DSS treatment for 7 days. DNase I solutions were freshly prepared in sterile saline (B. Braun Medical, Shanghai, China) prior to each injection.

### Metabolomics profiling of *Lactobacillus johnsonii* N5 CFS

To characterize the small-molecule metabolites in *L. johnsonii* N5 CFS, untargeted metabolomics was performed. Two biological replicates of CFS (each pooled from three independent supernatant preparations) and two replicates of sterile MRS broth (control), were stored in liquid N and analyzed via liquid chromatography-mass spectrometry (LC-MS) using an Agilent 6530 Q-TOF mass spectrometer coupled to a 1200 HPLC system (Agilent Technologies, Santa Clara, CA). Chromatographic separation was achieved using a reversed-phase column (e.g., ZORBAX Eclipse Plus C18, 2.1 × 100 mm, 1.8 μm) under conditions described previously^56,57^.

MSconvert was used to convert raw LC-MS data files from vendor-specific formats to generic mzXML format^58^. Untargeted feature detection and alignment were performed using XCMS, which operated in R (v4.3.1) for statistical analysis. Compound Discoverer software was used for LC-MS/MS data processing, which mainly included a series of analyses such as peak extraction, peak alignment and metabolite identification. The data exported by Compound Discoverer were used for data pre-processing, QC analysis, and subsequent analysis through metaX.

### Sample collection

At the terminal endpoint (day 7, Fig. 1A), mice were euthanized by *i.p*. injection of 200 μL of 0.9% sodium pentobarbital solution. A total of ten fecal samples (*n* = two cages per group) were obtained for baseline microbiota analysis prior to treatment initiation. Blood was collected via cardiac puncture under terminal anesthesia using EDTA-coated tubes. Serum was prepared by centrifugation at 2000 × *g* for 20 min at 4 °C and stored at −80 °C until analysis. Colon length was measured. Feces and luminal contents from the colon were snap-frozen in liquid nitrogen and stored at −80 °C. Tissues from the distal colon, liver, spleen and kidney were obtained and divided, where sections were made for histological, immunohistochemistry and immunofluorescence microscopy examination. While fresh tissues were immediately processed for various analyses or frozen in liquid nitrogen, and stored at −80 °C. Tissues (distal colon, liver, spleen, kidney) were dissected and divided into aliquots. For histology/immunohistochemistry and immunofluorescence, samples were fixed in 4% paraformaldehyde (PFA) or embedded in OCT compound.

### Enzyme-linked immunosorbent assay (ELISA)

The concentrations of colonic pro-inflammatory cytokines (IL-1β, IL-6 and IL-8) were measured using ELISA kits (Beyotime, Shanghai, China), according to the manufacturer’s instructions. The levels of MPO in distal colonic tissues were measured using ELISA kit (EMMPO, Thermo Fisher Scientific, China) following the manufacturer’s instructions. Briefly, tissue samples were homogenized and protein levels were measured in the homogenates with the Bio-Rad DC Protein Assay (Bio-Rad Laboratories, Shanghai, China). Values were normalized to tissue protein levels. Moreover, fecal and seral NGAL levels were quantified using a mouse NGAL ELISA Kit (CSB-E09410m, Cusabio, China).

In addition, ELISA analysis of MPO–DNA Complex was conducted to quantify NETs in isolated neutrophil cell culture. First, 5 μg/mL of anti-MPO monoclonal antibody (PA5-16672, Thermo Fisher Scientific), used as the capturing antibody, was coated to a 96-well plate overnight at 4 °C. After blocking with 1% BSA, samples and the peroxidase-labeled anti-DNA monoclonal antibody (1:25, 11774425001, Roche, Switzerland) were added to each well and incubated for 2 h at 37 °C and was washed three times. Finally, peroxidase substrate was added. The optical density (OD) of each well was measured at a wavelength of 450 nm (OD_450_). The results were expressed as mean OD values.

### Histological analysis and Alcian blue/periodic acid–Schiff staining

For histological analysis, distal colon samples were fixed in 4% PFA, processed and embedded in paraffin, sectioned at 4 µm thickness and stained with hematoxylin and eosin (H&E) following the manufacturer’s protocol (G1120, Solarbio, China). Distal colonic sections were scored for mucosal damage according to a previously described method^5^: 0 (intact crypt), 1–3 (partial damage), or 4 (complete loss of crypt and epithelium), multiplied by the percentage of tissue involvement. Liver tissues were embedded in Tissue-Tek® OCT compound, sectioned at 10-μm thickness using a cryostat microtome (CM1950, Leica, Germany), and stained for further analysis. All sections were evaluated under a light microscope (Leica DMi8, Leica, Wetzlar, Germany). In addition, to identify goblet cells, distal colonic sections were stained with Alcian blue/periodic acid–Schiff (AB-PAS) (Solarbio, Beijing, China) according to the manufacturer’s instructions. PAS^+^ goblet cells were counted in 10 longitudinally sectioned crypts and expressed as cells per crypt.

### Quantitative RT PCR

Total RNA was extracted from liver and distal colonic tissues using TRIzol Reagent (Invitrogen, 15596026, Waltham, MA, USA) and quantified with a NanoDrop 1000 spectrophotometer (F-3100, Suizhen, Hangzhou, China). One μg of RNA was reverse-transcribed into cDNA using HiScript II Q RT SuperMix (Vazyme biotech, R222-01, Nanjing, China) according to the manufacturer’s protocol. Quantitative RT-PCR (qRT-PCR) analysis was performed using AceQ qPCR SYBR Green Master Mix (Vazyme Biotech, Q111-02, Nanjing, China) on an ABI QuantStudio 3 Real-Time PCR Instrument. Gene expression levels (fold change) were normalized to the internal reference *Gapdh* and calculated using the 2^−ΔΔCT^ method. Primer sequences were as follows: *il1b*, forward 5’-*CTAAACAGATGAAGTGCTCC*-3’, reverse 5’-*GGTCATTCTCCTGGAAGG*-3’; *il6*, forward 5’-*ATGAACTCCTTCTCCACAAGCGC*-3’, reverse 5’-*GAAGAGCCCTCAGGCTGGACTG*-3’; *cxcl8*, forward 5’-*TGTAAACATG*-*ACTTCCAAGC*-3’, reverse 5’-*AAAACTGCACCTTCACAC*-3’; *Gapdh*, forward 5’-*GCAAGGATGCCCCAATGT*-3’, reverse 5’-*AGCAAGACAGTTGGTTGTGCAG*-3’.

### RNA extraction and transcriptomic analysis

The total RNA was extracted from colon and liver tissues and its concentration was measured using a NanoDrop 1000 spectrophotometer. Furthermore, an Agilent Bioanalyzer 2100 system (Agilent Technologies, CA, USA) was used to evaluate the quality of RNA. Thereafter, an Illumina TruSeq RNA Sample Prep Kit (Illumina, CA, USA) was used to construct the RNA-seq libraries. The libraries for the high-throughput sequencing were constructed using an Illumina HiSeq 2000 sequencer (BGI Tech, Wuhan, China). The sequencing data was filtered with SOAPnuke (v1.5.6) to obtain clean reads which were stored in FASTQ format. Bowtie2 (v2.5.0) was used to align high-quality clean reads to GRCm39. For subsequent data processing, cufflinks software was used to obtain quantitative fragments per kilobase of exon model per million mapped fragments (FPKM) values. Differential expression analysis was performed using the DESeq2 with the threshold differentially expressed for genes set at log2 (fold change)>1 and adjusted *P* < 0.05.

### DNA extraction and 16S rDNA amplicon sequencing

DNA was extracted from the fecal samples using the TIANamp Stool DNA Kit (TIANGEN, Beijing, China) according to the manufacturer’s instructions, and its concentration was measured using a NanoDrop 1000 spectrophotometer. Gut microbiota composition was characterized by 16S rRNA gene sequencing as described previously^29^. Briefly, the V3-V4 regions of bacterial 16S rRNA were amplified to construct DNA libraries for sequencing. Each reaction was subjected to PCR following assessment of concentration and purity. Fusion PCR primer mixtures, PCR premixes and 30 ng of genomic DNA were mixed in a 50 ml V3-V4 region PCR reactions buffer. The primer sequences amplified in the V3-V4 region were forward 5′-*ACTCCTACGGGGAGGCAGCAG*-3′ and reverse 5′-*GGACTACHVGGGTWTCTAAT*-3′, which were designed for PCR amplification with denaturation at 95 °C for 3 min; 30 cycles of 95 °C for 45 s, followed by annealing at 56 °C for 45 s; elongation at 72 °C for 45 s; and a final extension at 72 °C for 10 min. The AMPure XP magnetic beads were used to purify PCR products. The libraries fragment ranges and concentrations were examined using an Agilent 2100 Bioanalyzer. Finally, the validated libraries were sequenced as pair-ends on Illumina HiSeq platform (2 × 250 bp, Novogene, China). The Illumina sequencing data output was processed according to the cut-offs and pipeline described previously^59^. Sequence splicing was performed using the software FLASH (Fast Length Adjustment of Short reads, v1.2.11)^60^, which uses overlapping relationships to assemble pairs of reads obtained by double-end sequencing into a single sequence, and to obtain tags in the high-variable region. Usearch was used for clustering, clustering according to 97% sequence similarity to generate operational taxonomic units (OTUs)^61^. The OTU sequences were classified using the “sintax” algorithm with parameter “-sintax_cutoff 0.8” based on the UNITE (v8.3) database.

### In vivo permeability assay

Gut permeability/leakage was assessed by FITC-dextran assay as described previously^8^. Briefly, the mice were orally administered FITC-labeled dextran (MW 4000 DA; Sigma-Aldrich) at 60 mg/100 g body weight, with food and water withdrawn 4 h before. Blood was collected and serum FITC concentration was determined by fluoro-spectrometry (490/525 nm, Thermo Scientific) using standard curves generated by serial dilution of FITC-dextran with values from mice without the administration as background. Furthermore, serum endotoxin (lipopolysaccharide, LPS) levels were measured using ELISA (Pierce LAL Chromogenic Endotoxin Quantification Kit, Thermo Scientific) according to the manufacturer’s instructions.

### Organ tissue culture

Tissues from the liver, spleen, kidney and blood were harvested under sterile conditions for bacterial translocation investigations as previously described^62^. The samples were homogenized and 100 µL of the homogenate or blood was plated on blood agar and incubated at 37 °C with 5% CO_2_ for 1–3 days to detect bacteria growth.

### Immunofluorescent imaging

For immunofluorescence staining, tissue samples were cryosectioned at 10 µm thickness and fixed with pre-chilled acetone (−20 °C) for 20 min. Slides were permeabilized with 0.5% Triton X-100 (Sigma-Aldrich) in PBS for 15 min, blocked with 5% BSA for 30 min at room temperature, followed by staining for target proteins at 4 °C overnight in dark. The antibodies and dyes used included: UEA-1 lectin (L32476, ThermoFisher Scientific) and anti-Ki67 (ab15580, Abcam), anti-H3cit (ab5103, Abcam), anti-Ly6G (127603, BioLegend) and anti-CD41 (ab134131, Abcam) antibodies. After several washes with 0.05% Tween-20 in PBS, fluorescently labeled secondary antibodies (Alexa Fluor 488 anti-rat, Alexa Fluor 555 anti-rabbit, Alexa Fluor 555 anti-rat and Alexa Fluor 647 anti-rat IgG) were added and incubated at room temperature in the dark for 2 h. Nuclei were counterstained with Hoechst 33342 (Thermo Fisher Scientific).

Two slides per mouse (*n* = 6 per group) were imaged using a light microscope (Leica DMi8, Leica, Wetzlar, Germany) or a confocal Laser Scanning Microscope (Leica CS SP2, Wetzlar, Germany). For UEA-1^+^ mucin quantification, the percentage of positive area per crypt was measured in 10 random fields per slide using ImageJ software (Rasband, W.S., ImageJ, U.S. National Institutes of Health, Bethesda, Maryland, USA, https://imagej.nih.gov/ij/, 1997-2018). Ki67^+^ proliferative cells were counted in 10 longitudinally sectioned crypts and expressed as cells per crypt.

In livers, the Ly6G positive, H3cit positive and CD41 positive area (in tissue and in vessel) was evaluated in 10 random visions of one slide and mean area were calculated. In addition, fluorescent intensity of H3cit and CD41 were measured and background signals (vessel-associated for H3cit; hepatocyte-associated for CD41) were subtracted using ImageJ thresholding.

### Flow cytometry analysis

Fresh liver tissues were harvested, and single-cell suspensions were prepared by enzymatic tissue digestion with 20 mL of pre-warmed PBS containing 0.2 mg/mL collagenase II and IV, and 0.1 mg/mL DNAse I. and neutrophil populations were enriched by Percoll-based density gradient centrifugation and filtered through 70 μm cell strainers as previously described^12^. Isolation of leukocytes from blood samples were performed using buffy coat. Samples were blocked with anti-CD16/32 antibody (2.4G2, BD Bioscience) and stained with antibodies against CD45 (QA17A26), CD11b (M1/70), Ly6G (1A8) and CD41 (MWReg30) from BioLegend and CD162 (2PH1, BD Bioscience). Dead cells were excluded from analysis using the Live/Dead Aqua Viability Kit (Thermo Fisher Scientific) or 7AAD (BD Bioscience). For intracellular staining of H3cit (EPR17703, Abcam), cells were fixed and permeabilized using Fixation/Permeabilization Solution Kit (BD Cytofix/Cytoperm) according to the manufacturer’s instructions.

### In vitro NET induction and quantification

Neutrophils were isolated from mouse bone marrow as previously described^12^. Briefly, tibias and femurs were flushed with ice-cold HBSS (Invitrogen), bone debris and cell mass were removed by filtration through a cell sieve (70 μm) and the cell suspension was centrifuged (400 × *g*, 10 min). The pellet was resuspended in PBS, layered onto a discontinuous Percoll gradient (55%, 65%, 80%; Sigma-Aldrich), and centrifuged (1000 × *g*, 30 min). Neutrophils at the 65–80% interface were collected, washed in PBS, and subjected to hypotonic lysis to remove erythrocytes. Purified BMDNs were resuspended in HBSS (Invitrogen) at 1 × 106 cells/mL.

For NET induction, 2 × 10^5^ neutrophils were plated on coated coverslips, allowed to adhere for 1 h, and treated for 4 h at 37 °C with LPS (from *Escherichia coli* 055: B5, 5 μg/ml, Solarbio), LPS+DNase I (200 U/mL), LPS + *L. johnsonii* N5 (1 × 10^8^ CFU/mL), or LPS + CFS (N5 equivalent). All in vitro experiments were repeated independently at least six times to ensure reproducibility.

For confocal imaging, cells were fixed with 4% PFA (30 min, RT), blocked with 3% BSA (1 h, RT), and stained with anti-histone H3 (1:200, P63763-1B1S, Abmart, China), anti-MPO (1:100, ab208670, Abcam), and Hoechst 33342 (Invitrogen). Secondary antibodies (Alexa Fluor 488 donkey anti-rabbit, Alexa Fluor 647 donkey anti-goat, 1:500, Thermo Fisher) were applied for 1 h. Slides were mounted with ProLong Gold Antifade (Invitrogen) and imaged on a Leica stellaris 5 confocal microscope. NET releasing cells (NETosis) was quantified by measuring the area of MPO+/histone H3+ extracellular DNA structures in five random fields per sample using ImageJ, normalized to 100 cells.

### Statistics and reproducibility

Statistical analysis was carried out using GraphPad Prism version 9 (GraphPad Software, Inc., CA, USA) and R programming (version 4.3.3). Two-tailed Student’s *t* test or one-way analysis of variance (ANOVA) with Tukey’s post hoc test were used for normally distributed continuous variables. Changes in body weight and fecal scores over time were analyzed and compared using the area under the curve (AUC). Principal component analysis (PCA) based on OTUs abundance were conducted using the Bray–Curtis, unweighted, and weighted UniFrac distance metrics to visualize group differences. Permutational multivariate analysis of variance (PERMANOVA) was done using the adonis function in the vegan package of R (version 2.0-7) with default settings 999 permutations^63^. The GSEA tool (GSEA 4.1.0) was used for the Gene Set Enrichment Analysis to identify the significant enrichment pathways. Differences in the abundance of pathways were annotated using the Kyoto Encyclopedia of Genes and Genomes (KEGG) database based on STAMP software and Welch’s *t* test with Benjamini–Hochberg correction. Volcano plot analysis was performed using the OmicShare tools, a free online platform for data analysis (https://www.omicshare.com/tools). The package “VennDiagram” of R (version 4.3.3) was used to draw Venn diagram. Data were presented as mean ± SEM and significant differences were evaluated by Tukey’s multiple comparison tests; *p* < 0.05 was considered significant.

### Reporting summary

Further information on research design is available in the Nature Portfolio Reporting Summary linked to this article.

## Supplementary information

Supplementary Information

Description of Additional Supplementary Materials

Supplementary Data 1

Supplementary Data 2

Reporting Summary

## Data Availability

The 16S rRNA gene sequence data are available in the Sequence Read Archive (SRA) under BioProject accession numbers PRJNA1176198, PRJNA1253907 and PRJEB12149. RNA-seq data are available in the SRA under BioProject accession number PRJNA1175388. Metabolomic data of *L. johnsonii* N5 cell-free supernatant are available in figshare under 10.6084/m9.figshare.29634929, and other numerical source data for the current study can be found in Supplementary Data 2.
