# Supplementary figures and images for: Lactobacillus johnsonii alleviates experimental colitis by restoring intestinal barrier function and reducing NET-mediated gut-liver inflammation
Source: Commun Biol. 2025 Aug 14;8:1222. doi: 10.1038/s42003-025-08679-4 (PMC12354853; doi:10.1038/s42003-025-08679-4)

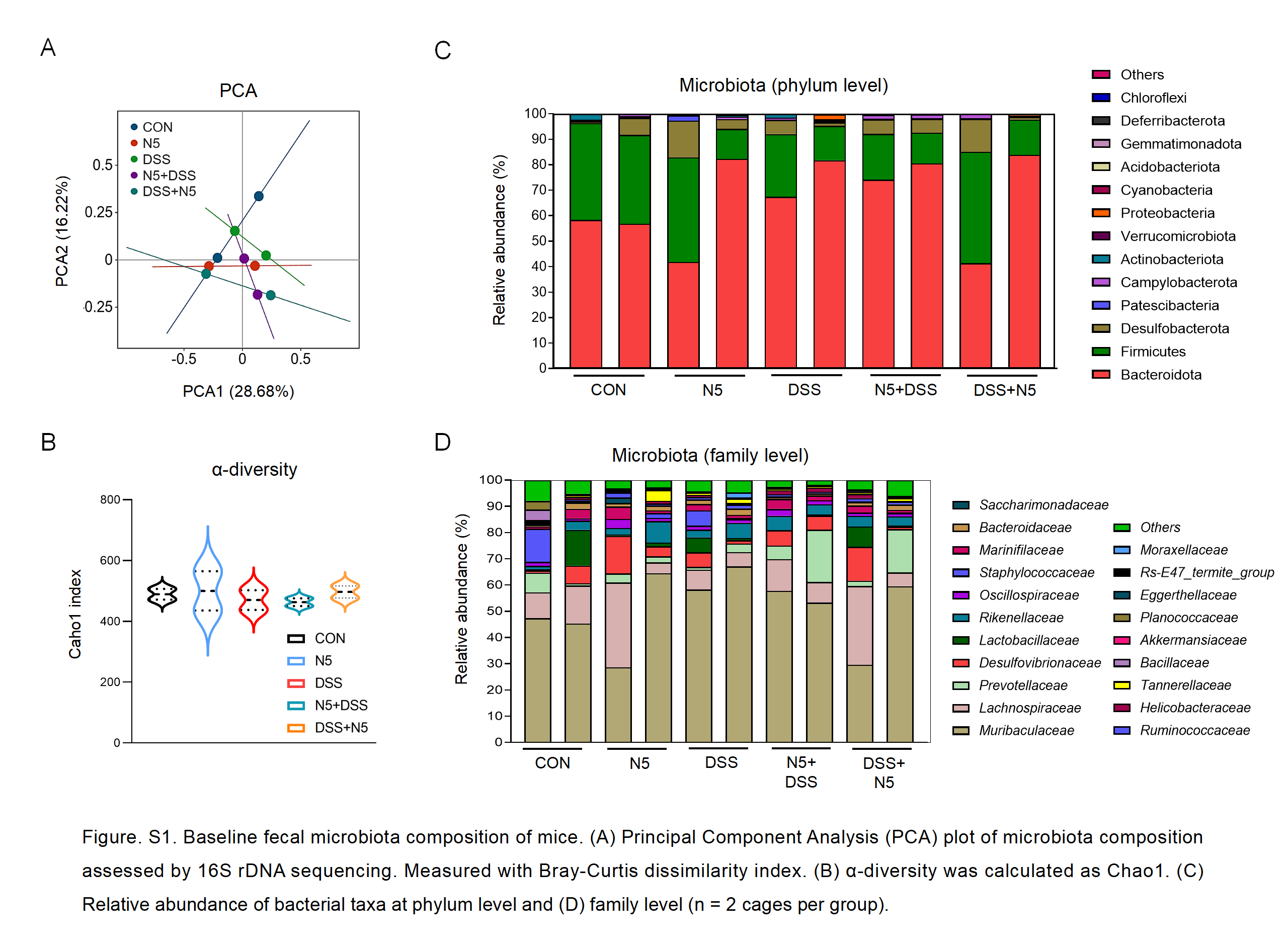

Supplement: Supplementary file 1 — Supplementary Information [file 42003_2025_8679_MOESM1_ESM.tif]
