# Supplementary material for: Lactobacillus johnsonii alleviates experimental colitis by restoring intestinal barrier function and reducing NET-mediated gut-liver inflammation
Source: Commun Biol. 2025 Aug 14;8:1222. doi: 10.1038/s42003-025-08679-4 (PMC12354853; doi:10.1038/s42003-025-08679-4)
Supplement: Supplementary file 2 — Description of Additional Supplementary Materials [file 42003_2025_8679_MOESM2_ESM.docx]

**Description of Additional Supplementary Files**

**File name:** Supplementary Data 1

**Description:** Metabolome data table of Lactobacillus johnsonii N5

**File name:** Supplementary Data 2

**Description:** Source data for figures
