# Supplementary material for: Lactobacillus johnsonii alleviates experimental colitis by restoring intestinal barrier function and reducing NET-mediated gut-liver inflammation
Source: Commun Biol. 2025 Aug 14;8:1222. doi: 10.1038/s42003-025-08679-4 (PMC12354853; doi:10.1038/s42003-025-08679-4)
Supplement: Supplementary file 5 — Reporting Summary [file 42003_2025_8679_MOESM5_ESM.pdf]

nature portfolio

Corresponding author(s): Demin Cai

Last updated by author(s): 2025 - 06 - 03

Reporting Summary

Nature Portfolio wishes to improve the reproducibility of the work that we publish. This form provides structure for consistency and transparency in reporting. For further information on Nature Portfolio policies, see our Editorial Policies and the Editorial Policy Checklist.

Statistics

For all statistical analyses, confirm that the following items are present in the figure legend, table legend, main text, or Methods section.

n/a	Confirmed	
☐	☒	The exact sample size (*n*) for each experimental group/condition, given as a discrete number and unit of measurement
☐	☒	A statement on whether measurements were taken from distinct samples or whether the same sample was measured repeatedly
☐	☒	The statistical test(s) used AND whether they are one- or two-sided<br>*Only common tests should be described solely by name; describe more complex techniques in the Methods section.*
☐	☒	A description of all covariates tested
☐	☒	A description of any assumptions or corrections, such as tests of normality and adjustment for multiple comparisons
☐	☒	A full description of the statistical parameters including central tendency (e.g. means) or other basic estimates (e.g. regression coefficient) AND variation (e.g. standard deviation) or associated estimates of uncertainty (e.g. confidence intervals)
☒	☐	For null hypothesis testing, the test statistic (e.g. *F*, *t*, *r*) with confidence intervals, effect sizes, degrees of freedom and *P* value noted<br>*Give P values as exact values whenever suitable.*
☒	☐	For Bayesian analysis, information on the choice of priors and Markov chain Monte Carlo settings
☒	☐	For hierarchical and complex designs, identification of the appropriate level for tests and full reporting of outcomes
☐	☒	Estimates of effect sizes (e.g. Cohen's *d*, Pearson's *r*), indicating how they were calculated

Our web collection on statistics for biologists contains articles on many of the points above.

Software and code

Policy information about availability of computer code

Data collection	no software was used
Data analysis	Statistical analysis was carried out using GraphPad Prism version 9 (GraphPad Software, Inc., CA, USA) and R programming (version 4.3.3).Permutational multivariate analysis of variance (PERMANOVA) was done using the adonis function in the vegan package of R (version 2.0-7) with default settings 999 permutations.The GSEA tool (GSEA 4.1.0) was used for the Gene Set Enrichment Analysis to identify the significant enrichment pathways. Differences in the abundance of pathways were annotated using the Kyoto Encyclopedia of Genes and Genomes (KEGG) database based on STAMP software and Welch's t-test with Benjamini-Hochberg correction. Volcano plot analysis was performed using the OmicShare tools, a free online platform for data analysis (https://www.omicshare.com/tools). The package "VennDiagram" of R (version 4.3.3) was used to draw Venn diagram.

For manuscripts utilizing custom algorithms or software that are central to the research but not yet described in published literature, software must be made available to editors and reviewers. We strongly encourage code deposition in a community repository (e.g. GitHub). See the Nature Portfolio guidelines for submitting code & software for further information.

Data

Policy information about availability of data

All manuscripts must include a data availability statement. This statement should provide the following information, where applicable:

- Accession codes, unique identifiers, or web links for publicly available datasets
- A description of any restrictions on data availability
- For clinical datasets or third party data, please ensure that the statement adheres to our policy

The 16S rRNA gene sequence data are available in the Sequence Read Archive (SRA) under BioProject accession numbers PRJNA1176198, PRJNA1253907 and PRJEB12149. RNA-seq data are available in the SRA under BioProject accession number PRJNA1175388.

Research involving human participants, their data, or biological material

Policy information about studies with human participants or human data. See also policy information about sex, gender (identity/presentation), and sexual orientation and race, ethnicity and racism.

Reporting on sex and gender	not applicable
Reporting on race, ethnicity, or other socially relevant groupings	not applicable
Population characteristics	not applicable
Recruitment	not applicable
Ethics oversight	not applicable

Note that full information on the approval of the study protocol must also be provided in the manuscript.

Field-specific reporting

Please select the one below that is the best fit for your research. If you are not sure, read the appropriate sections before making your selection.

☒ Life sciences ☐ Behavioural & social sciences ☐ Ecological, evolutionary & environmental sciences

For a reference copy of the document with all sections, see nature.com/documents/nr-reporting-summary-flat.pdf

Life sciences study design

All studies must disclose on these points even when the disclosure is negative.

Sample size	The sample size was determined using a power - analysis method. We aimed to detect a medium - sized effect (Cohen's d = 0.5) with a significance level (α) of 0.05 and a statistical power (1 - β) of 0.8.This calculation takes into account the expected effect size, the desired level of significance, and the power of the test. Adequate sample size is crucial as it ensures that the study has sufficient statistical power to detect true effects if they exist, reducing the risk of Type II errors (failing to reject a false null hypothesis).
Data exclusions	No data was excluded from the analyses. All samples that met the basic quality - control criteria were included in the final analysis.
Replication	The experiment was replicated three times independently. Each replication involved using a new batch of mice sourced from the same vendor and maintaining consistent experimental conditions, including housing, diet, and treatment protocols.
Randomization	Mice were randomly assigned to different groups using a computerized random number generator. This procedure ensures that each mouse has an equal probability of being allocated to any group, thereby minimizing potential selection bias.
Blinding	A double - blind design was employed. The researchers who administered L. johnsonii N5 and those who collected and analyzed the data were unaware of which mice belonged to the treatment or control groups. This was to prevent any potential observer bias in data collection and analysis.

Reporting for specific materials, systems and methods

We require information from authors about some types of materials, experimental systems and methods used in many studies. Here, indicate whether each material, system or method listed is relevant to your study. If you are not sure if a list item applies to your research, read the appropriate section before selecting a response.

Materials & experimental systems

n/a	Involved in the study	
☐	☒	Antibodies
☐	☒	Eukaryotic cell lines
☒	☐	Palaeontology and archaeology
☐	☒	Animals and other organisms
☒	☐	Clinical data
☒	☐	Dual use research of concern
☒	☐	Plants

Methods

n/a	Involved in the study	
☒	☐	ChIP-seq
☐	☒	Flow cytometry
☒	☐	MRI-based neuroimaging

Antibodies

Antibodies used: anti-MPO monoclonal antibody (PA5-16672, Thermo Fisher Scientific); peroxidase-labeled anti-DNA monoclonal antibody (1:25, 11774425001, Roche, Switzerland); anti-Ki67 (ab15580, Abcam), anti-H3cit (ab5103, Abcam), anti-Ly6G (127603, BioLegend) and anti-CD41 (ab134131, Abcam) antibodies; anti-CD16/32 antibody (2.4G2, BD Bioscience); CD45 (QA17A26), CD11b (M1/70), Ly6G (1A8) and CD41 (MWReg30) from BioLegend and CD162 (2PH1, BD Bioscience); anti-histone H3 (1:200, P63763-1B1S, Abmart, China), anti-MPO (1:100, ab208670, Abcam), and Hoechst 33342 (Invitrogen)

Validation: All the above antibodies were primary antibodies and the reacting host was mouse

Eukaryotic cell lines

Policy information about cell lines and Sex and Gender in Research

Cell line source(s): The neutrophil-enriched cell population was isolated from the bone marrow of wild-type male BALB/c mice.

Authentication: Neutrophils were verified by immunofluorescence

Mycoplasma contamination: This cell line was not contaminated

Commonly misidentified lines (See ICLAC register): No commonly misidentified cell lines were used in this study. The isolation and identification procedures were designed to specifically target and confirm the identity of mouse bone marrow neutrophils, reducing the risk of misidentification.

Animals and other research organisms

Policy information about studies involving animals; ARRIVE guidelines recommended for reporting animal research, and Sex and Gender in Research

Laboratory animals: Wild-type male BALB/c mice (8–10 weeks old)

Wild animals: No wild animals were involved in the experiment

Reporting on sex: The results were used for any mouse sex

Field-collected samples: Jiangsu Laboratory Animal Science Center and housed under specific pathogen-free conditions (22 °C, 50% humidity, 12/12-h light/dark cycle) with free access to water and standard chow

Ethics oversight: Animal experiments in this study were performed following the guidelines of the Animal Care and Use Committee of Yangzhou University [SYXK (SU) 2021-0026]

Note that full information on the approval of the study protocol must also be provided in the manuscript.

Plants

Seed stocks: not applicable

Novel plant genotypes: not applicable

Authentication: not applicable

Flow Cytometry

Plots

Confirm that:

☒ The axis labels state the marker and fluorochrome used (e.g. CD4-FITC).

☒ The axis scales are clearly visible. Include numbers along axes only for bottom left plot of group (a 'group' is an analysis of identical markers).

☒ All plots are contour plots with outliers or pseudocolor plots.

☒ A numerical value for number of cells or percentage (with statistics) is provided.

Methodology

Sample preparation	Fresh liver tissues were harvested, and single-cell suspensions were prepared by enzymatic tissue digestion with 20 mL of pre-warmed PBS containing 0.2 mg/mL collagenase II and IV, and 0.1 mg/mL DNAse I. and neutrophil populations were enriched by Percoll-based density gradient centrifugation and filtered through 70µm cell strainers as previously described.Isolation of leukocytes from blood samples were performed using buffy coat.
Instrument	The data was collected using a BD FACSCanto II flow cytometer (BD Biosciences, Franklin Lakes, NJ, USA).
Software	FlowJo software (Tree Star, Inc., Ashland, OR, USA) was used for data collection and analysis. Custom code was not used in this study.
Cell population abundance	After sorting, the purity of the neutrophil population was approximately 90% as determined by flow cytometry. This was achieved by comparing the percentage of cells expressing specific markers within the sorted fraction. The abundance of the neutrophil population in the sorted fraction was calculated from the total number of cells counted by flow cytometry.
Gating strategy	For the starting cell population, the preliminary forward scatter (FSC) and side scatter (SSC) gates were set to exclude cell debris and aggregates.

☒ Tick this box to confirm that a figure exemplifying the gating strategy is provided in the Supplementary Information.
